# Dynamics of a Mathematical Model for Tuberculosis with Variability in Susceptibility and Disease Progressions Due to Difference in Awareness Level

**DOI:** 10.3389/fmicb.2015.01530

**Published:** 2016-01-26

**Authors:** Daniel Okuonghae, Bernard O. Ikhimwin

**Affiliations:** Department of Mathematics, University of BeninBenin City, Nigeria

**Keywords:** mathematical model, tuberculosis, awareness level, bifurcation, simulation

## Abstract

This work extends a mathematical model for the transmission dynamics of tuberculosis that examined the impact of certain factors on tuberculosis case detection (Okuonghae and Omosigho, 2011). The extended model now classifies the latently infected individuals by their level of tuberculosis awareness (as was done for the susceptible sub-population) and further expands the number of key factors that can positively affect the tuberculosis case detection rate. The effect of these identified factors on the associated reproduction number of the model is considered. It is shown that the system can undergo the phenomenon of backward bifurcation when the associated reproduction number of the model is less than unity; in a special case, the effect of exogenous re-infection on the backward bifurcation phenomenon is significantly dictated by the level of awareness of the latently infected individuals. Qualitative and quantitative analysis of the model showed the effect of key identified factors on the dynamics of tuberculosis while suggesting a serious concentration on tuberculosis awareness programmes, active case finding strategies and use of active cough identification for identifying likely TB cases and sustaining awareness campaigns over a long period of time.

## 1. Introduction

Tuberculosis (TB), an infectious disease caused by the *Mycobacterium tuberculosis* bacillus, remains one of the world's deadliest diseases (World Health Organization, 2014). According to the World Health Organization (WHO), in 2013, about 9 million people were infected, worldwide, with TB and 1.5 million deaths from the disease were reported, 360,000 of whom were HIV-positive (World Health Organization, 2014). Tuberculosis is seen to be declining slowly each year and an estimated 37 million lives were saved between 2000 and 2013 through effective diagnosis and treatment (World Health Organization, 2014). On the average, TB incidence fell to about 1.5% per year, between 2000 and 2013, worldwide (World Health Organization, 2014). Globally, TB mortality rate fell by an estimated 45% between 1990 and 2013 while the prevalence rate dropped by 41% (World Health Organization, 2014). Even with such positive results achieved within the last 14 years, it is still thought that deaths from tuberculosis are preventable; in fact the death toll is still considered unacceptably high. Hence, efforts are geared toward accelerating programmes that will result in a reduction in the TB burden globally [within the context of the Millennium Development Goals (MDGs)], and reach the Stop TB Partnership target of a 50% reduction by 2015 (World Health Organization, 2014).

More than half of the approximately 9 million individuals who are infected with tuberculosis in 2013 (56%) were in South-East Asia and Western Pacific Regions. A further one quarter of these infected individuals are in the African Region, which account for the highest rates of TB cases and deaths relative to population (World Health Organization, 2014).

Generally, reducing the incidence and prevalence of TB in a population hinges on successful treatment and high case detection rates (Okuonghae and Omosigho, 2010, 2011; World Health Organization, 2014; Okuonghae, 2015). It fact, it is seriously encouraged that effort should be concentrated on ensuring that all TB cases are detected, notified and commence treatment immediately (World Health Organization, 2014). It is reported that about 6.1 million TB cases were reported to the WHO in 2013 out of which about 5.7 million were individuals were newly diagnosed and another 0.4 million were already on treatment (World Health Organization, 2014). Tuberculosis notification has stabilized in recent years, with about 64% of the estimated 9 million individuals who developed TB in 2013 were notified as newly diagnosed cases (World Health Organization, 2014). This implies that about 3 million cases were either not diagnosed, or diagnosed but not reported to national TB programmes (World Health Organization, 2014) which could hinder the goal of significantly reducing the prevalence of TB in such localities. Treatment success rates (globally) have been impressive (and continue to be high) over the years, with about 86% treatment success rate reported in 2013 among all new TB cases (World Health Organization, 2014).

Tuberculosis affects family and social relationships and results in adverse health and economic consequences (Armijos et al., 2008; Chang and Cataldo, 2014). As stated earlier, improving the case detection and notification rates will result in reducing the TB burden in a population. However, several factors could be hindering efforts at improving these rates. Individuals infected with TB and their families can experience prejudice and negative attitudes, such as shame, blame and a sense of judgment as a result of the infection (Bennstam et al., 2004; Baral et al., 2007; Chang and Cataldo, 2014). Stigmatization can also be a stumbling block in improving the tuberculosis case detection rate (Johansson et al., 2000; Okuonghae and Omosigho, 2010; Chang and Cataldo, 2014) since patients and families' fears of inferiority stems from the anticipation of an adverse judgement related to a TB diagnosis (Johansson et al., 2000; Chang and Cataldo, 2014).

A survey reported in Okuonghae and Omosigho (2010) listed some factors that can adversely affect the implementation of the directly observed treatment, short-course (DOTS) strategy in Nigeria (one of the high burden countries) in reducing the incidence of TB in the country. The survey revealed that most persons do not know how TB is transmitted and the signs and symptoms of tuberculosis; several individuals are not even aware of the government's health policies on tuberculosis and TB treatment. Further, the survey revealed that this lack of awareness can lead to delays in reporting likely TB cases for treatment (Okuonghae and Omosigho, 2010), increasing the likelihood of disease transmission.

Analysis of the results from the survey (Okuonghae and Omosigho, 2010) identified four key factors that must be combined for an effective control of tuberculosis: “effective awareness programme, active cough identification, associated cost factor for treatment of identified cases and effective treatment” (Okuonghae and Omosigho, 2010).

A mathematical model for TB dynamics that incorporated the identified factors (as parameters) gleaned from the work in Okuonghae and Omosigho (2010) was formulated and analyzed in Okuonghae and Omosigho (2011). Control strategies, based on the identified parameters, that can lead to minimizing the incidence (as well as the prevalence) of the disease in a population were proposed. In summary, the qualitative and quantitative analysis of the model in Okuonghae and Omosigho (2011) showed that a serious concentration on tuberculosis awareness programmes and active cough identification as a marker for identifying potential TB cases can be significant in minimizing the severity of the disease in a population, with effective treatment.

The purpose of this article is to present and analyze a new mathematical model for TB dynamics; this model is an extension of that presented and analyzed in Okuonghae and Omosigho (2011). The aim of this work is to further study the effects of additional heterogeneities based on the level of awareness of tuberculosis within the population and active-case finding, on the dynamics of the disease. In the work in Okuonghae and Omosigho (2011), only the susceptible subpopulation was stratified by their level of tuberculosis awareness. In this work, we will now stratify both the susceptible and latently infected sub-populations by their level of awareness of TB (symptoms and signs of TB as well as testing and treatment programmes available by the government). Note that the measure of the case detection and notification rates will be by the number of infectious individuals detected, notified, and treated for tuberculosis.

## 2. Materials and methods

### 2.1. Basic model

This section briefly describes the model in Okuonghae and Omosigho (2011). In Okuonghae and Omosigho (2011), we assumed that susceptible individuals are divided into two groups depending on their level of awareness of the disease (and any treatment policy): the high risk (low level of awareness) group, *S*_1_, and the “educated,” low risk (high level of awareness) group, *S*_2_. The *S*_1_ class is “educated” at the per capita rate α_1_ and thereafter move into the *S*_2_ class. Tuberculosis infection can invade the *S*_1_ and *S*_2_ classes, depending on the “efficacy” of the education programme. The programme is assumed to reduce the likelihood of infection by a factor of σ (0 ≤ σ ≤ 1). The case σ = 0 signifies a completely effective education program, while σ = 1 models the situation where the program is totally ineffective.

It was further assumed that the “vaccine” (education program) produces temporary immunity at the per capita rate θ. The case θ = ∞ corresponds to the case where there is absolutely no immunity while θ = 0 corresponds to life-long immunity. Hence, θ measures the rate at which those in the *S*_2_ class return to the *S*_1_ class due to forgetfulness caused by lack of continuous exposure to the enlightenment program while the disease persists in the community. We assumed β to be the disease transmission rate.

We also assumed that the variables *E*, *I*, *J*, and *T* represented the “primary” latent, infectious, identified infectious (for treatment under DOTS) and the effectively treated individuals, respectively (by “primary” latency, we are referring to susceptible individuals who are infected for the first time as well as treated individuals who now get reinfected after recovering from a previous infection). In addition to these groups, a separate class (*R*(*t*)) was added to account for individuals who become latent due to failed treatment or self cure (we refer to this situation as “secondary” latency).

The parameter η was used as a modification parameter (0 ≤ η ≤ 1) to account for the relative infectiousness of infectious individuals in the *J* class. We also assumed that ϵ is the reduced likelihood of reinfection of effectively treated individuals, where 0 ≤ ϵ ≤ 1. Also, we took 0 < *p* < 1 as the fraction of individuals with new infections who develop TB fast per unit of time, with Λ being the rate of recruitment of uninfected newborns and immigrants into the low risk susceptible class. For simplifications, we assumed that all entrants into the population move into the *S*_1_ group. We also assumed that μ is the natural death rate while *d* is the tuberculosis-induced death rate.

To account for exogenous reinfection of latently infected individuals, we assumed that β^*^ be the transmission rate amongst this group in infected persons. We also assumed that *k* is the rate of progression of infected individuals in the latent stage to active tuberculosis.

Individuals with active TB can be identified using chronic cough lasting more than 2 weeks as a marker, at the rate α_2_, and are referred to a TB treatment program under DOTS for effective treatment (in Okuonghae and Omosigho, 2011, α_2_ was known as the cough identification rate). However, a fraction of these identified cases will eventually get into the treatment program when we consider the cost factor. Hence, a cost improvement factor (ν : 0 < ν ≤ 1) will affect the actual number of identified cases that commences treatment. The cost factor considers the effect the actual cost of medical tests and treatment will have on the care givers when presenting the infectious individual for treatment. If ν = 0, then the cost of medical tests and treatment is prohibitively high and να_2_ = 0 implies that the TB case will not get into a TB treatment program due to the financial cost on the care givers or family members. However, if ν = 1, it means that the cost of medical tests and treatment is totally free and να_2_*I* will be the total number of identified cases that are tested and treated for tuberculosis under DOTS. Hence, να_2_ was taken to be the proportion of identified TB cases that commences treatment under DOTS. Since in most developing countries, like Nigeria, it was observed that medical tests for TB still cost money especially when the infectious person is about commencing treatment (Okuonghae and Omosigho, 2010), it then implies that ν ≠ 1; it lies in the range 0 < ν < 1. In all, ν → 1 implies that the cost of testing and treating TB becomes affordable.

We assumed that *r*_2_ is the treatment rate for the identified infectious individuals under the DOTS scheme while the fraction of the detected cases who were successfully treated under the DOTS was *n*, with *m* = 1 − *n* being the fraction of those whose treatment were unsuccessful and, thereafter, moved to the “secondary” latency group.Tuberculosis cases that are not detected either die at the rate *d*, or self-cure and revert to the “secondary” latent state (in *R*) at the rate *r*_1_.

The mathematical model was then given by the following system of non-linear ordinary differential equations (Okuonghae and Omosigho, 2011):

(1a) $$\frac{dS_{1}}{dt}\;=\Lambda -\alpha_{1}S_{1}-\beta S_{1}\frac{\left(I+\eta J\right)}{N}+\theta S_{2}-\mu S_{1},$$

(1b) $$\frac{dS_{2}}{dt}=\alpha_{1}S_{1}-\sigma \beta S_{2}\frac{\left(I+\eta J\right)}{N}-\theta S_{2}-\mu S_{2},$$

(1c) $$\begin{array}{l} \frac{dE}{dt}=(1-p)(\beta S_{1}\frac{\left(I+\eta J\right)}{N}+\sigma \beta S_{2}\frac{\left(I+\eta J\right)}{N}+\epsilon \beta T\frac{\left(I+\eta J\right)}{N})\\ \;-\beta^{*}E\frac{\left(I+\eta J\right)}{N}-(k+\mu )E, \end{array}$$

(1d) $$\begin{array}{l} \frac{dI}{dt}=p(\beta S_{1}\frac{\left(I+\eta J\right)}{N}+\sigma \beta S_{2}\frac{\left(I+\eta J\right)}{N}+\epsilon \beta T\frac{\left(I+\eta J\right)}{N})+kE\\ \;+\beta^{*}E\frac{\left(I+\eta J\right)}{N}-(\nu \alpha_{2}+\mu +d+r_{1})I, \end{array}$$

(1e) $$\frac{dJ}{dt}=\nu \alpha_{2}I-r_{2}J-\mu J,$$

(1f) $$\frac{dT}{dt}=nr_{2}J-\mu T-\epsilon \beta T\frac{\left(I+\eta J\right)}{N},$$

(1g) $$\frac{dR}{dt}=r_{1}I+mr_{2}J-\mu R.$$

The effective reproduction number of model (1) is given as

(2) $$\begin{array}{l} R_{c}=\frac{\beta }{d+\mu +r_{1}+\nu \alpha_{2}}\frac{\left(k+p\mu \right)}{k+\mu }\frac{\left(\mu +r_{2}+\eta \nu \alpha_{2}\right)}{\mu +r_{2}}\\ \;\left[\frac{\mu +\theta }{\mu +\alpha_{1}+\theta }+\frac{\sigma \alpha_{1}}{\mu +\alpha_{1}+\theta }\right] \end{array}$$

See Okuonghae and Omosigho (2011) and Okuonghae (2015) for the qualitative and quantitative analysis of model (1) and the effect of the key parameters, gleaned from the survey in Okuonghae and Omosigho (2010), on the dynamics of TB in a population.

### 2.2. Modified mathematical model

**Figure 1:**
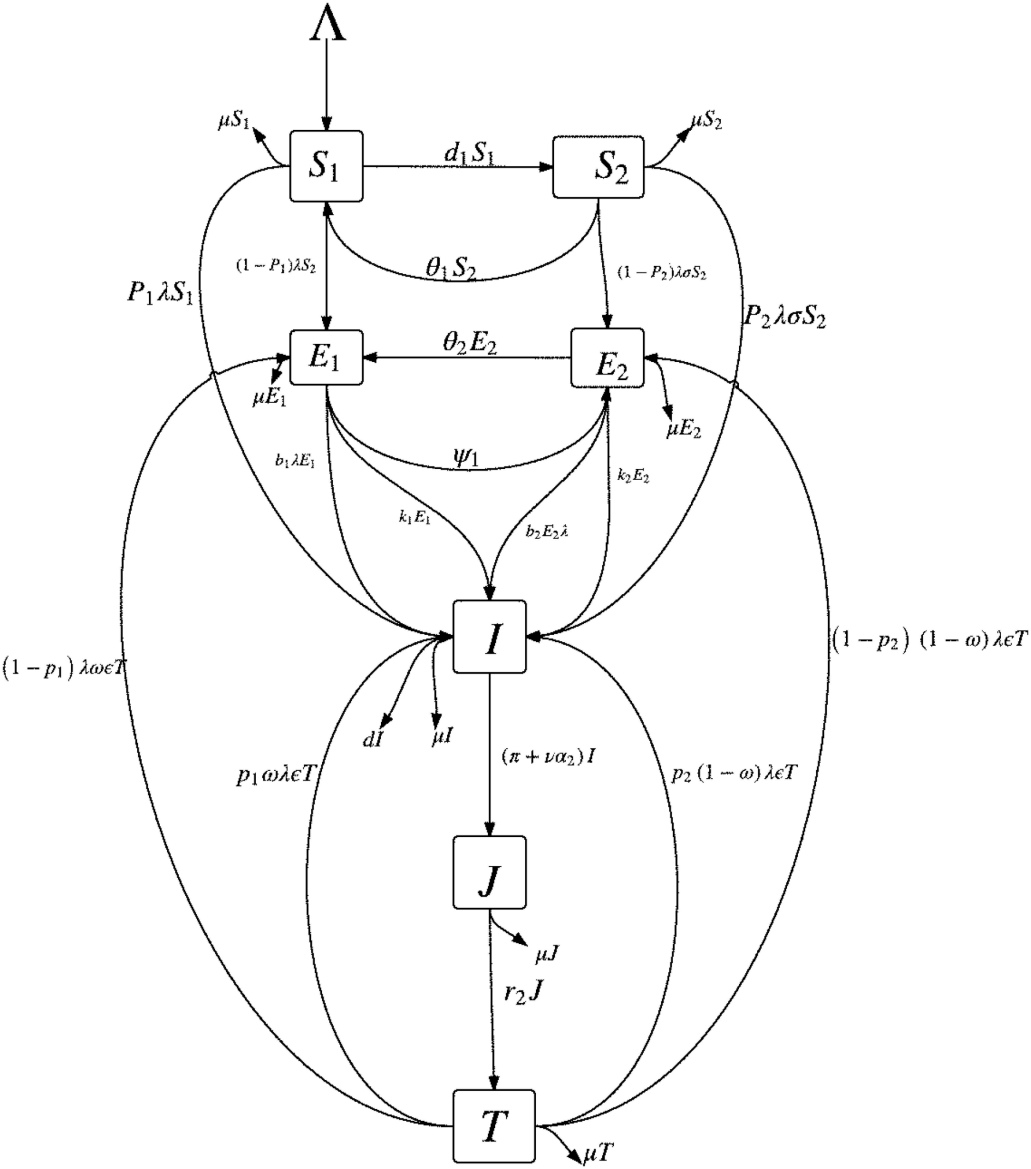
**Schematic diagram of model given in (3), where $\lambda =\frac{\beta \left(I+\eta J\right)}{N}$**.

Instead of stratifying only the susceptible subpopulation by their level of awareness (i.e., *S*_1_ and *S*_2_ as described in Section 2.1), we will also stratify the latently infected class by their level of awareness. This is reasonable since latently infected individuals do not transmits TB (they do not show the signs and symptoms of TB) and, in most cases, will become aware of their disease status only when tuberculin tests are carried out on them; for example, the Mantoux tuberculin skin test (TST). Figure 1 shows the schematic diagram of the modified mathematical model given in (3).

In addition to some of the epidemiological classes in Section 2.1, we will break up the latent class (*E*) into two classes, depending on their level of awareness of the disease: the latently infected, high risk (low level of awareness) group, *E*_1_, and the “educated” latently infected, low risk (high level of awareness) group, *E*_2_. It can be stated that due to awareness, individuals in the *E*_2_ group are tested in order to know their disease status and take necessary precautionary health measures. The *E*_1_ class is made up of individuals from the *S*_1_ class and some individuals from the treated class (*T*) who have a low level of awareness of tuberculosis while the *E*_2_ class is made up of individuals from the *S*_2_ class and some persons from the treated class (*T*) who retained their high level of awareness following their recovery. The latently infected individuals with low awareness (*E*_1_) can become “educated” at the per capita rate ψ and thereafter move into the *E*_2_. Individuals who recover (following effective treatment) can get reinfected, with a fraction 0 ≤ ω ≤ 1 of such persons entering the class of latent individuals with low level of awareness (*E*_1_) while the remaining fraction 1 − ω enter the *E*_2_ class.

We also assume that the education programmes produces “temporary immunity” at the per capita rate θ_1_ (for the susceptible groups, *S*_1_ and *S*_2_) and at the rate θ_2_ (for the latent groups, *E*_1_ and *E*_2_). The cases θ_1_(θ_2_) = ∞ corresponds to the situation where there is absolutely no immunity (from the education programmes) while θ_1_(θ_2_) = 0 corresponds to life-long immunity. Hence, θ_1_(θ_2_) measures the rate at which those in the *S*_2_(*E*_2_) class return to the *S*_1_(*E*_1_) class due to a lack of continuous exposure to the awareness programmes while the disease persists in the community. We further assume that β is the disease transmission rate.

Also, we assume that 0 < *p*_1_(*p*_2_) < 1 represent the fraction of persons with new infections who develop TB fast per unit of time from the class of infected individuals with low level of awareness (high level of awareness). We expect that, by virtue of the benefits of awareness, *p*_1_ ≥ *p*_2_ as new infections are quickly detected and fewer cases of fast progressions to active TB are recorded amongst individuals with a high level of awareness; also, note that the period of fast latency can span between 1 and 5 years (Styblo, 1980; Flynn and Chan, 2001; Colijn et al., 2007) so that early detection can prevent more cases of active TB.

We further assume that *b*_1_(*b*_2_) are modification parameters accounting for exogenous re-infection of the latently infected individuals in the *E*_1_(*E*_2_) class, with 0 ≤ *b*_2_ ≤ *b*_1_ < 1 and that *k*_1_(*k*_2_) is the rate of progression of the individuals in the latent state (*E*_1_(*E*_2_)) to active tuberculosis. Assume that, in addition, to the impact of active cough identification (α_2_) and the cost factor (ν) on improving the case detection (and notification) rates, evident by the number of infectious individuals in the *J* class (and treatment), let π be the rate at which an active case-finding strategy is used in searching for infectious TB cases for onward treatment with the treatment rate now represented as *r*. The remaining parameters in the modified model are as defined in Section 2.1. Since there is significant improvement in treatment success rate for TB (worldwide) (World Health Organization, 2014), especially in countries and communities, where there is an efficient treatment strategy put in place (for example, DOTS), we assume an insignificant number of failed treatments and self cure and will omit the *R* class from the modified model.

On the basis of the above assumptions, the modified model is now given by the following system of non-linear ordinary differential equations:

(3a) $$\frac{dS_{1}}{dt}=\Lambda -\alpha_{1}S_{1}-\beta S_{1}\frac{\left(I+\eta J\right)}{N}+\theta_{1}S_{2}-\mu S_{1},$$

(3b) $$\frac{dS_{2}}{dt}=\alpha_{1}S_{1}-\sigma \beta S_{2}\frac{\left(I+\eta J\right)}{N}-\theta_{1}S_{2}-\mu S_{2},$$

(3c) $$\begin{array}{l} \frac{dE_{1}}{dt}=(1-p_{1})\left(\beta S_{1}\frac{\left(I+\eta J\right)}{N}+\omega \epsilon \beta T\frac{\left(I+\eta J\right)}{N}\right)\\ \;-b_{1}\beta E_{1}\frac{\left(I+\eta J\right)}{N}-(k_{1}+\mu +\psi )E_{1}+\theta_{2}E_{2}, \end{array}$$

(3d) $$\begin{array}{l} \frac{dE_{2}}{dt}=(1-p_{2})\left(\sigma \beta S_{2}\frac{\left(I+\eta J\right)}{N}+(1-\omega )\epsilon \beta T\frac{\left(I+\eta J\right)}{N}\right)\\ \;-b_{2}\beta E_{2}\frac{\left(I+\eta J\right)}{N}-(k_{2}+\mu +\theta_{2})E_{2}+\psi E_{1}, \end{array}$$

(3e) $$\begin{array}{l} \frac{dI}{dt}=p_{1}\beta (S_{1}+\omega \epsilon T)\frac{\left(I+\eta J\right)}{N}+p_{2}\beta (\sigma S_{2}\\ \;+(1-\omega )\epsilon T)\frac{\left(I+\eta J\right)}{N}+\beta (b_{1}E_{1}+b_{2}E_{2})\frac{\left(I+\eta J\right)}{N}\\ \;+k_{1}E_{1}+k_{2}E_{2}-(\nu \alpha_{2}+\mu +d+\pi )I, \end{array}$$

(3f) $$\frac{dJ}{dt}=(\pi +\nu \alpha_{2})I-rJ-\mu J,$$

(3g) $$\frac{dT}{dt}=rJ-\mu T-\epsilon \beta T\frac{\left(I+\eta J\right)}{N},$$

with *N* = *S*_1_ + *S*_2_ + *E*_1_ + *E*_2_ + *I* + *J* + *T*.

#### 2.2.1. Basic properties of model (3)

For model (3) to be epidemiologically meaningful, it is important to prove that all its state variables are non-negative for all time *t*. In other words, the solutions of model (3) with positive initial data will remain positive for all time *t* ≥ 0.

Theorem 2.1. *Let the initial data for model* (3) be *S*_1_(0) > 0, *S*_2_(0) > 0, *E*_1_(0) > 0, *E*_2_(0) > 0, *I*(0) > 0, *J*(0) > 0 *and T*(0) > 0. *Then, the solutions*

$$\left(S_{1}(t),S_{2}(t),E_{1}(t),E_{2}(t),I(t),J(t),T(t)\right)$$

*of model* (3), *with positive initial data, will remain positive for all time t* > 0.

Theorem 2.2. *The closed set*

$$D=\left\{(S_{1},S_{2},E_{1},E_{2},I,J,T)\in {\mathbb{R}}_{+}^{7};N\leq \frac{\Lambda }{\mu }\right\}$$

*is positively invariant and attracts all positive solutions of model* (3).

See Appendix A for the proofs of Theorems 2.1 and 2.2.

Since the region D is positively invariant, the unique solution of model (3) exists and depends continuously on the initial data of the model (hence, it is sufficient to study its asymptotic dynamics in the region D, Hethcote, 2000).

#### 2.2.2. Local asymptotic stability of disease-free equilibrium

Model (3) has a disease-free equilibrium (DFE), obtained by setting the right hand sides of the equations in model (3) to zero and solve for the state variables with no infections, given by

$$\begin{array}{l} \xi_{1}=(S_{1}^{*},S_{2}^{*},E_{1}^{*},E_{2}^{*},I^{*},J^{*},T^{*})\\ \;=\left(\frac{\Lambda }{\mu }\frac{\mu +\theta_{1}}{\mu +\alpha_{1}+\theta_{1}},\frac{\Lambda }{\mu }\frac{\alpha_{1}}{\mu +\alpha_{1}+\theta_{1}},0,0,0,0,0\right) \end{array}$$

We see that the susceptible classes, at the DFE, depend on a factor of the asymptotic population size, $\frac{\Lambda }{\mu }$.

The local stability of ξ_1_ can be established with the next generation operator method on system (3) (Diekmann et al., 1990; van den Driessche and Watmough, 2002). Using the notations in van den Driessche and Watmough (2002), it follows that matrices *F* and *V*, for the new infection terms and the remaining transition terms, respectively, are given by

$$F=\left(\begin{array}{ccccc} 0 & 0 & (1-p_{1})\beta \frac{S_{1}^{*}}{N^{*}} & (1-p_{1})\beta \eta \frac{S_{1}^{*}}{N^{*}} & \\ 0 & 0 & (1-p_{2})\beta \sigma \frac{S_{2}^{*}}{N^{*}} & (1-p_{2})\beta \eta \sigma \frac{S_{2}^{*}}{N^{*}} & \\ 0 & 0 & \beta \frac{\left(p_{1}S_{1}^{*}+p_{2}\sigma S_{2}^{*}\right)}{N^{*}} & \beta \eta \frac{\left(p_{1}S_{1}^{*}+p_{2}\sigma S_{2}^{0}\right)}{N^{*}} & 0\\ 0 & 0 & 0 & 0 & \end{array}\right)$$

and

$$V=\left(\begin{array}{cccc} \left(k_{1}+\mu +\psi \right) & \text{​​}-\theta_{2} & 0 & 0\\ \text{​​}-\psi & \text{​​}(k_{2}+\mu +\theta_{2}) & 0 & 0\\ \text{​​}k_{1} & -k_{2} & \text{​​}(\nu \alpha_{2}+d+\mu +\pi ) & 0\\ \text{​​}0 & 0 & -(\pi +\nu \alpha_{2}) & \text{​​}(r+\mu )\text{​​} \end{array}\right)\text{​}.$$

Thus, the effective reproduction number of model (3), denoted by R_*T*_, is given by

(4) $$R_{T}=\frac{\beta (\mu +\eta \pi +r+\eta \nu \alpha_{2})[G_{1}+G_{2}+G_{3}]}{G_{4}},$$

where

$$\begin{array}{l} G_{1}=k_{1}[(\mu +\sigma \alpha_{1}+\theta_{1})(k_{2}+\theta_{2})+\mu (\mu +\sigma \alpha_{1}p_{2}+\theta_{1})],\\ G_{2}=\mu (\mu +\theta_{2}+\psi )[\sigma \alpha_{1}p_{2}+p_{1}(\mu +\theta_{1})],\\ G_{3}=k_{2}(\mu +\theta_{1})(\mu p_{1}+\psi )+k_{2}\sigma \alpha_{1}(\mu +\psi ),\\ G_{4}=(\mu +r)(d+\mu +\pi +\nu \alpha_{2})(\mu +\alpha_{1}+\theta_{1})[(\mu +k_{1})\\ \;(\mu +k_{2}+\theta_{2})+(\mu +k_{2})\psi ], \end{array}$$

and R_*T*_ is obtained from ρ(*FV*^−1^) with ρ being the spectral radius of the matrix *FV*^−1^.

The following result follows from Theorem 2 in (van den Driessche and Watmough, 2002):

Lemma 2.1. *The DFE*, ξ_1_, *of model (3) is locally asymptotically stable if* R_*T*_ < 1 *and unstable if* R_*T*_ > 1.

The threshold quantity, R_*T*_, represents the average number of secondary tuberculosis infections generated by a typical infectious individual in a completely susceptible population with already existing controls such as treatment (Hethcote, 2000; van den Driessche and Watmough, 2002). The epidemiological implication of Lemma 2.1 is that tuberculosis can be effectively controlled in the community (when R_*T*_ < 1) if the initial sizes of the subpopulation of model (3) are in the basin of attraction of the DFE, ξ_1_, in the presence of control strategies including chronic cough identification, active-case finding, effective cost factor, effective awareness programmes and treatment. Hence, a small influx of individuals with active TB into the community will not generate large TB outbreaks, and the disease will die out with time.

#### 2.2.3. Comparing reproduction numbers under different control scenarios

It is important to compare different scenarios involving the presence of control measures based on the reproduction number for the different situations.

**Case 1**:

Re-write the effective reproduction number as

(5) $$R_{T}=R_{F}\frac{\mu (\pi \eta +\mu +r+\eta \nu \alpha_{2})}{\left(\pi \eta +\mu +\eta \nu \alpha_{2})(\mu +r\right)}\equiv R_{F}A_{1}$$

where

$$A_{1}=\frac{\mu (\pi \eta +\mu +r+\eta \nu \alpha_{2})}{\left(\pi \eta +\mu +\eta \nu \alpha_{2})(\mu +r\right)}$$

and R_*F*_ = R_*T*_|_*r* = 0_ is the reproduction number of model (3), under control, without treatment (where R_*T*_|_*r* = 0_ is the effective reproduction number, with *r* = 0).

Since the difference of R_*F*_ from R_*T*_ is only in treatment, the factor *A*_1_ compares a population with and without treatment; however other control strategies such as chronic cough identification (with an effective cost factor) and active-case finding are present in the community. We observe that *A*_1_ will be less than unity (*A*_1_ < 1) for all 0 ≤ η, ν < 1.

Generally, if R_*F*_ < 1, then we cannot expect a TB epidemic and in this case no treatment strategy is needed for control. However, when R_*F*_ > 1, we need to determine the necessary condition for slowing the development of tuberculosis in the community. Following Mukandavire et al. (2007); Sharomi et al. (2008); Okuonghae and Omosigho (2011), we have the difference between R_*F*_ and R_*T*_ as

(6) $$\Delta_{T}:=R_{F}-R_{T}=(1-A_{1})R_{F}.$$

For effective treatment, use of chronic cough as a marker for potential TB cases (with an effective cost factor) and an effective active case-finding strategy to slow down the spread of tuberculosis in a population, we expect that Δ_*T*_ > 0, and this condition is satisfied if *A*_1_ < 1 in Equation (6). Now, setting R_*T*_ = 1 and solving for *A*_1_, we have the threshold effectiveness of treatment and cough identification taking into account the cost factor and an active case-finding programme:

(7) $$A_{1}^{*}=\frac{1}{R_{F}}$$

Hence, tuberculosis can be eradicated from the population if the control measures include effective treatment, active chronic cough identification (taking into cognisance an effective cost factor) and an active case-finding strategy if $A_{1}<A_{1}^{*}$. Observe from Equation (7) that $A_{1}^{*}$ is a decreasing function of R_*F*_; higher values of $A_{1}^{*}$ results in lower values of R_*F*_, a desired outcome.

Taking the following limits of *A*_1_ provides further insight into possible ways of reducing the TB burden in a community:

(8) $$(i)\underset{\begin{array}{c} \nu \to 1\\ \alpha_{2}\to \infty \end{array}}{\lim}A_{1}\;=\underset{\begin{array}{c} \pi \to \infty \\ \nu \to 1\\ \alpha_{2}\to \infty \end{array}}{\lim}A_{1}=\underset{\begin{array}{c} \pi \to \infty \\ \nu \to 1\\ \alpha_{2}\to \infty \\ \eta \to 0 \end{array}}{\lim}A_{1}=\frac{\mu }{\mu +r},$$

(9) $$(ii)\underset{r\to \infty }{\lim}A_{1}\;=\underset{\begin{array}{c} r\to \infty \\ \nu \to 1 \end{array}}{\lim}A_{1}=\frac{\mu }{\mu +\pi \eta +\eta \alpha_{2}},(iii)\underset{\begin{array}{c} r\to \infty \\ \eta \to 0 \end{array}}{\lim}A_{1}=1.$$

Observe that the limits of *A*_1_ in Equations (8) and (9)(ii) are less than one; hence control strategies for tuberculosis gleaned from these results can be pursued, if it is feasible and practicable economically. In practice, ν → 1 implies near total elimination of costs (medical tests and treatment), α_2_ → ∞ implies high rate of identifying “potential” TB cases using chronic cough as a marker, *r* → ∞ implies high treatment rate, π → ∞ implies a high active case-finding rate for infectious TB individuals and η → 0 implies that those identified for treatment have a significantly reduced likelihood of transmitting the disease.

Using the factor *A*_1_, one observes that an effective combination of chronic cough identification (with the associated cost factor), an active case-finding strategy with effective treatment and taking care to prevent detected infectious TB cases, receiving treatment, from causing new TB infections, will result in reducing the burden of TB in the population.

It is very interesting to observe that more than one combination of control strategies could yield the same positive, desired result. For example, as seen in the limits of *A*_1_ in Equation (8), concentrating on using chronic cough as a marker for a potential TB case with little cost involved in testing and treatment is as effective as including an effective active case-finding strategy to the aforementioned strategy. Also, an effective combination of treatment rate and cost factor can also lead to a reduction in the number of new TB infections in the population.

**Case 2**:

Consider the situation where there are no control strategies put in place for detecting active TB cases via the use of chronic cough identification and an active case-finding strategy so that π = α_2_ = ν = 0. Clearly then, we observe that (*J, T*) → 0 asymptotically, as *t* → ∞, in model (3). Therefore, the effective reproduction number of the model (3), with π = α_2_ = ν = 0, R_*B*_, is written as

$$R_{B}=R_{T}{|}_{\pi =\alpha_{2}=\nu =0}.$$

Now, we have that R_*T*_ = *A*_2_R_*B*_ where

$$A_{2}=\frac{d+\mu }{d+\pi +\mu +\nu \alpha_{2}}\frac{\pi \eta +\mu +r+\eta \nu \alpha_{2}}{\mu +r}.$$

Here, *A*_2_ is known as the *effectiveness of treatment, chronic cough identification (with an effective cost factor), an active case-finding strategy, and reduced infectivity of isolated infectious cases undergoing treatment* on the control of tuberculosis.

If R_*B*_ < 1, then TB cannot develop into an epidemic and no control strategy involving treatment, chronic cough identification (with a cost factor) and an active case-finding strategy is needed for TB control. If we take the difference between R_*B*_ and R_*T*_ i.e.,

$$\Delta_{B}:=R_{B}-R_{T}=(1-A_{2})R_{B}$$

then an effective combination of the above-mentioned controls for slowing down the spread of TB in the population (as stated above in the expression for *A*_2_) would mean that Δ_*B*_ > 0, which is satisfied if *A*_2_ < 1.

It is very interesting to note the expressions for the limits of *A*_2_, when the limits applied to *A*_1_ are used, namely:

(10) $$(i)\underset{\begin{array}{c} \nu \to 1\\ \alpha_{2}\to \infty \end{array}}{\lim}A_{2}=\underset{\begin{array}{c} \pi \to \infty \\ \nu \to 1\\ \alpha_{2}\to \infty \end{array}}{\lim}A_{2}=\underset{\begin{array}{c} \nu \to 1\\ r\to \infty \end{array}}{\lim}A_{2}=\frac{\eta (d+\mu )}{\mu +r},$$

(11) $$\begin{array}{l} (ii)\underset{r\to \infty }{\lim}A_{2}=\underset{\begin{array}{c} r\to \infty \\ \eta \to 0 \end{array}}{\lim}A_{2}=\frac{d+\mu }{d+\mu +\pi +\nu \alpha_{2}}\text{and}\\ (iii)\underset{\begin{array}{c} \pi \to \infty \\ \eta \to 0\\ \nu \to 1\\ \alpha_{2}\to \infty \end{array}}{\lim}A_{2}=0. \end{array}$$

Other than the limits of *A*_2_ in Equation (11) (iii), the results of the other limits of *A*_2_ in Equations (10) and (11) (ii) are adjusted by disease-induced death and are less than unity, when compared to the corresponding limits for *A*_1_, as discussed earlier. Interestingly, the limit in Equation (11) (ii) is zero, compared to the same limit that gave $\frac{\mu }{\mu +r}$, for *A*_1_; hence, the former have a lesser value in the limit compared to the latter.

**Case 3**

In this case, we are considering the situation where there are no awareness programmes for both susceptible and latently infected individuals. However, we assume that treated individuals now have a measure of awareness following their treatment and recovery from TB. Hence, we have that α_1_ = ψ = θ_1_ = θ_2_ = 0. We observe, from model (3) that, for this case, *S*_2_ → 0 asymptotically, as *t* → ∞, so that the latently infected class *E*_2_ is now populated by treated individuals who now have some level of awareness, probably due to the experience they went through during the course of their treatment. We assume that those who are now populating the *E*_2_ class have life-long awareness with life long benefit (so that θ_2_ = 0). Hence, the effective reproduction number of the model (3), with α_1_ = ψ = θ_1_ = θ_2_ = 0, R_*A*_, is written as

$$R_{A}=R_{T}{|}_{\alpha_{1}=\psi =\theta_{1}=\theta_{2}=0}.$$

Now, we have that R_*T*_ = *A*_3_R_*A*_ where

$$A_{3}=\frac{B_{1}}{B_{2}},$$

with *B*_1_ = (μ(μ + *k*_1_)(πη + μ + *r* + η*να*_2_)(*k*_1_(μ(μ + σα_1_*p*_2_ + θ_1_) + (μ + σα_1_ + θ_1_)(*k*_2_ + θ_2_)) + σ*k*_2_α_1_(μ + ψ) + *k*_2_(μ + θ_1_)(μ*p*_1_ + ψ) + μ(σα_1_*p*_2_ + *p*_1_(μ + θ_1_))(μ + θ_2_ + ψ))) and *B*_2_ = [(*k*_1_ + μ*p*_1_)(μ + *r*)(πη + μ + η*να*_2_)(μ + α_1_ + θ_1_)[(μ + *k*_1_)(μ + *k*_2_ + θ_2_) + (μ + *k*_2_)ψ]].

In this case, *A*_3_ is known as the *effectiveness of treatment, chronic cough identification in the presence of a cost improvement factor, an active case-finding strategy, reduced infectivity of isolated infectious cases (for treatment) and awareness program for both susceptible and latent individuals* on the control of tuberculosis.

If R_*A*_ < 1, then TB cannot develop into an epidemic and no control strategy involving the aforementioned factors (as stated in the preceding paragraph) is needed for TB control.

If we take the difference between R_*A*_ and R_*T*_ i.e.,

$$\Delta_{A}:=R_{A}-R_{T}=(1-A_{3})R_{B},$$

then an effective combination of the controls (as mentioned above and expressed in *A*_3_) for slowing down the spread of TB in the population would mean that Δ_*A*_ > 0, which is satisfied if *A*_3_ < 1.

Taking some limits of *A*_3_ threw up some interesting conclusions as to effectively controlling tuberculosis, in this case:

(12) $$(i)\underset{\begin{array}{c} r\to \infty \\ \alpha_{1}\to \infty \\ \theta_{1}\to 0\\ \pi \to \infty \end{array}}{\lim}A_{3}=\underset{\begin{array}{c} \pi \to \infty \\ r\to \infty \\ \psi \to \infty \\ \theta_{2}\to 0 \end{array}}{\lim}A_{3}=\underset{\begin{array}{c} \nu \to 1\\ r\to \infty \\ \pi \to \infty \\ \theta_{2}\to 0\\ \theta_{1}\to 0\\ \psi \to \infty \\ \alpha_{1}\to \infty \\ \alpha_{2}\to \infty \end{array}}{\lim}A_{3}=0,$$

(13) $$(ii)\underset{\begin{array}{c} \alpha_{1}\to \infty \\ \psi \to \infty \\ \theta_{1}\to 0\\ \theta_{2}\to 0 \end{array}}{\lim}A_{3}=\frac{\mu \sigma (\mu +k_{1})(k_{2}+p_{2}\mu )(\pi \eta +\mu +r+\eta \nu \alpha_{2}}{\left(\mu +k_{2})(k_{1}+p_{1}\mu )(\mu +r)(\pi \eta +\mu +\eta \nu \alpha_{2}\right)},$$

and

(14) $$(iii)\underset{\begin{array}{c} \nu \to 1\\ \pi \to \infty \\ \theta_{2}\to 0\\ \theta_{1}\to 0\\ \psi \to \infty \\ \alpha_{1}\to \infty \\ \alpha_{2}\to \infty \end{array}}{\lim}A_{3}=\frac{\mu \sigma (\mu +k_{1})(k_{2}+p_{2}\mu )}{\left(\mu +k_{2})(k_{1}+p_{1}\mu )(\mu +r\right)}.$$

Clearly, the limits of *A*_3_ that includes *r* (treatment rate) are zero, and this is a positive outcome. However, for the limits of *A*_3_ in Equations (13) and (14), the limiting values of *A*_3_ will be zero if σ → 0 i.e., the situation where susceptible individuals who have a high awareness level have very reduced likelihood of getting infected with tuberculosis due to their level of awareness and the benefits that accrue from such awareness. Clearly, effective control strategies for tuberculosis that utilizes strategies associated with the expression for *A*_3_ will assist in reducing the TB burden in a community as can be seen from the results in Equation (12).

**Case 4**:

We will now discuss the situation where there are no disease controls in the system. In this worse case scenario, we will set *r* = ν = α_2_ = π = α_1_ = ψ = θ_1_ = θ_2_ = 0. This implies that, from model (3), (*S*_2_, *E*_2_, *J, T*) → 0 asymptotically, as *t* → ∞. Hence the **basic reproduction number** is obtained as

$$R_{0}=R_{T}{|}_{r=\nu =\alpha_{2}=\pi =\alpha_{1}=\psi =\theta_{1}=\theta_{2}=0}.$$

So that R_*T*_ = *A*_4_R_0_ where

$$A_{4}=\frac{D_{1}}{D_{2}},$$

with *D*_1_ = ((*d* + μ)(μ + *k*_1_)(πη + μ + *r* + η*να*_2_)(*k*_1_(μ(μ + σα_1_*p*_2_ + θ_1_) + (μ + σα_1_ + θ_1_)(*k*_2_ + θ_2_)) + σ*k*_2_α_1_(μ + ψ) + *k*_2_(μ + θ_1_)(μ*p*_1_ + ψ) + μ(σα_1_*p*_2_ + *p*_1_(μ + θ_1_))(μ + θ_2_ + ψ))) and *D*_2_ = [(*k*_1_ + μ*p*_1_)(μ + *r*)(*d* + π + μ + να_2_)(μ + α_1_ + θ_1_)[(μ + *k*_1_)(μ + *k*_2_ + θ_2_) + (μ + *k*_2_)ψ]].

If R_0_ < 1, then TB cannot develop into an epidemic and no control strategy is needed for TB control. If we take the difference between R_0_ and R_*T*_ i.e.,

$$\Delta_{0}:=R_{0}-R_{T}=(1-A_{4})R_{0}$$

then an effective combination of the controls discussed in this case will slow down the spread of TB in the population, which would mean that Δ_0_ > 0, which is satisfied if *A*_4_ < 1.

Applying the limits in Case 3 on *A*_4_, we observe that the results are the same except for

(15) $$(i)\underset{\begin{array}{c} \alpha_{1}\to \infty \\ \psi \to \infty \\ \theta_{1}\to 0\\ \theta_{2}\to 0 \end{array}}{\lim}A_{4}=\frac{\left(d+\mu )\sigma (\mu +k_{1})(k_{2}+p_{2}\mu )(\pi \eta +\mu +r+\eta \nu \alpha_{2}\right)}{\left(\mu +k_{2})(k_{1}+p_{1}\mu )(\mu +r)(d+\pi +\mu +\nu \alpha_{2}\right)}$$

and

(16) $$(ii)\underset{\begin{array}{c} \nu \to 1\\ \pi \to \infty \\ \theta_{2}\to 0\\ \theta_{1}\to 0\\ \psi \to \infty \\ \alpha_{1}\to \infty \\ \alpha_{2}\to \infty \end{array}}{\lim}A_{4}=\frac{\eta (d+\mu )\sigma (\mu +k_{1})(k_{2}+p_{2}\mu )}{\left(\mu +k_{2})(k_{1}+p_{1}\mu )(\mu +r\right)}$$

The difference is that the limits of *A*_4_ are disease-induced death adjusted (compared to the limits of *A*_3_) and, in Equations (15) and (16), the limiting results for *A*_4_ will be zero if σ → 0 or η → 0 [η → 0 applies just for Equation (16)] i.e., susceptible individuals who have a high awareness level have very reduced likelihood of getting infected with tuberculosis due to their level of awareness (and the benefits that accrue therefrom) and infectious individuals detected for treatment also have a reduced likelihood of infecting susceptible and latently infected individuals with tuberculosis during the course of their treatment.

Of course, the above results (discussed in Cases 1 to 4, in this section) are dependent on the fact that the baseline populations are not subjected to the same interventions. For example, in Case 1, *A*_1_ measures treatment effectiveness for a population where education, chronic cough identification and an active case-finding strategy are already implemented (without treatment) while in Case 4, *A*_4_ measures the effort to reduce the reproduction number for a population that had no intervention whatsoever; hence in the latter case (Case 4), the effort to curtail the disease must be higher than in the former (Case 1).

In summary, parameter values that would make *A*_1_ < 1, *A*_2_ < 1, *A*_3_ < 1 or *A*_4_ < 1 could yield control strategies with the capacity of reducing the number of secondary TB infections and slow down the spread of tuberculosis in the population. Hence, to determine the necessary condition for slowing down the development of TB at the population level, we will require that Δ_*F*_ > 0, Δ_*B*_ > 0, Δ_*A*_ > 0 or Δ_0_ > 0, as the case may be and, appropriately, determine control scenarios that will give better results in reducing the incidence (and prevalence) of tuberculosis in the population.

It is imperative to state that there are several limits that could be considered while studying *A*_1_, *A*_2_, *A*_3_ and *A*_4_. However, our interests are in investigating control strategies involving the combination of the following parameters: ν, π, ψ, θ_1_, θ_2_, α_2_, *r*, α_1_ and η. The combined effect of these parameters on control measures and their effect on on the dynamics of tuberculosis is worth examining and tested in improving the case detection rate as well as reduce the tuberculosis burden in a population.

#### 2.2.4. Analysis of effective reproduction number under controls, R_*T*_

Using the threshold parameter, R_*T*_, we want to study the effect of treatment, active cough identification and the associated cost factor, tuberculosis awareness, an active case-finding technique as well as a combination of some of these factors on the dynamics of TB in the population.

It is evident from (4) that

(17) $$\underset{\begin{array}{c} \alpha_{1}\to \infty \\ \psi \to \infty \\ \theta_{1}\to 0\\ \theta_{2}\to 0 \end{array}}{\lim}R_{T}=\frac{\beta \sigma (k_{2}+p_{2}\mu )(\pi \eta +\mu +r+\eta \nu \alpha_{2})}{\left(k_{2}+\mu )(\mu +r)(d+\pi +\mu +\nu \alpha_{2}\right)}>0,$$

(18) $$\underset{\begin{array}{c} \alpha_{1}\to \infty \\ \psi \to \infty \\ \theta_{1}\to 0\\ \theta_{2}\to 0\\ r\to \infty \end{array}}{\lim}R_{T}=\frac{\beta \sigma (k_{2}+p_{2}\mu )}{\left(k_{2}+\mu )(d+\pi +\mu +\nu \alpha_{2}\right)}>0,$$

(19) $$\underset{\begin{array}{c} \nu \to 1\\ \alpha_{2}\to \infty \\ \alpha_{1}\to \infty \\ \psi \to \infty \\ \theta_{1}\to 0\\ \theta_{2}\to 0\\ r\to \infty \end{array}}{\lim}R_{T}=\underset{\begin{array}{c} \alpha_{2}\to \infty \\ \nu \to 1\\ \pi \to \infty \\ r\to \infty \end{array}}{\lim}R_{T}=0.$$

It seems, from the limits of R_*T*_ in Equations (17)–(19), that a high treatment rate is very effective in the control of tuberculosis only when the effect of the other critical parameters are properly harnessed e.g., high awareness rates, less loss of awareness (forgetfulness), increased active case-finding and chronic cough identification (with minimal costs involved in testing and treatments).

**Figure 2:**
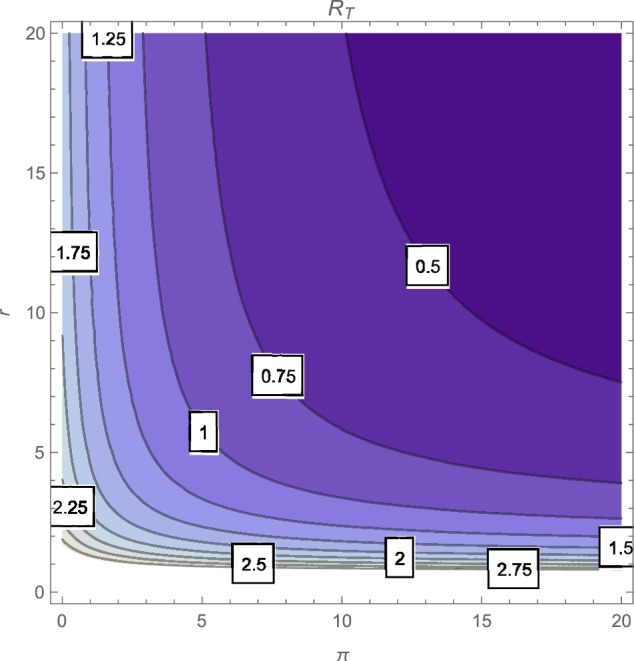
**Contour plot of R_*T*_ as a function of π and *r***.

**Figure 3:**
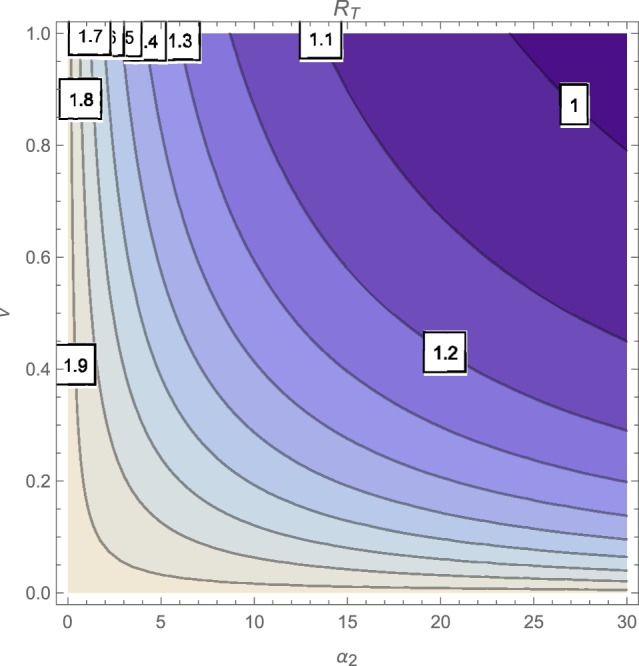
**Contour plot of R_*T*_ as a function of ν and α_2_**.

**Figure 4:**
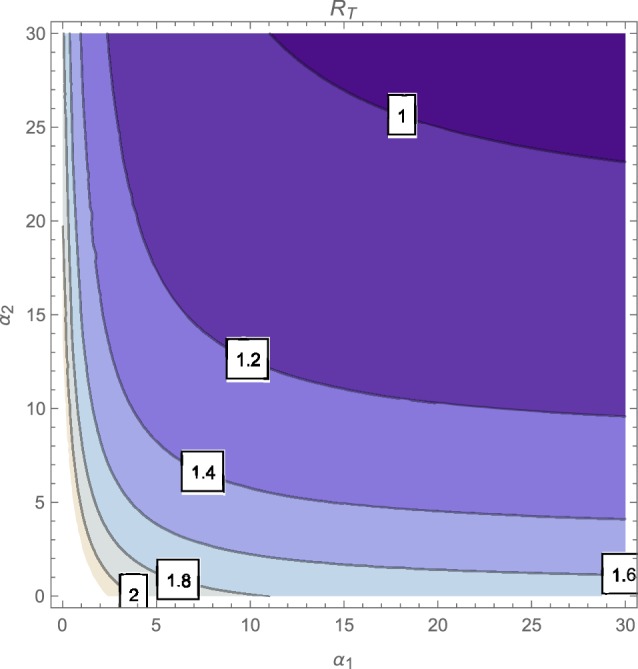
**Contour plot of R_*T*_ as a function of α_1_ and α_2_**.

The contour plot of R_*T*_, as a function of the active case-finding rate (π) and the treatment rate (*r*) is shown in Figure 2 while Figures 3, 4 depicts the contour plots of R_*T*_ as a function of the chronic cough identification rate (α_2_) and the associated cost factor (ν) and as a function of the awareness rate for the susceptible group (α_1_) and the chronic cough identification rate (α_2_), respectively. Using the base parameter values in Table 1, we observe from Figures 2–4 that the indicated parameters have a positive effect on the effective reproduction number, R_*T*_. Hence, improving on the awareness rate for the susceptible individuals, the associated cost factor, active case-finding strategy, the use of chronic cough as a TB marker (for likely cases) and effective treatment, will reduce the value of R_*T*_, albeit at different rates.

**Table 1:** **Parameter information**.

**Parameters**	**Description**	**Values**	**Units**
μ	natural death rate	0.02041 (0.0143, 0.04)	*year*^−1^
Λ	recruitment rate	μ × 10^5^	*year*^−1^
β	trans. rate	8.557 (4.4769, 15.1347)	*year*^−1^
*b*_1_, *b*_2_	trans. rate(exogenous re-infection)	0.2 (0, 1)	*year*^−1^
*p*_1_, *p*_2_	frac. of fast prog.	0.1 (0.05, 0.3)	*year*^−1^
*k*_1_, *k*_2_	prog. rate	0.05 (0.005, 0.05)	*year*^−1^
*r*	recovery rate	1.5 (1.5, 2.5)	*ind*^−1^*yr*^−1^
*d*	TB-induced death	0.365 (0.22, 0.39)	*year*^−1^
ν	cost factor	0.5 (0, 1)	*year*^−1^
η	mod. parameter	0.4 (0,1)	*year*^−1^
α_1_, ψ	awareness rate	5 (0,40)	*year*^−1^
α_2_	cough iden. rate	5 (0,40)	*year*^−1^
ϵ	reduce infec.	0.2 (0,1)	*year*^−1^
θ_1_, θ_2_	“immunity” measure	1 (0,40)	*year*^−1^
σ	effect. of program	0.5 (0,1)	*year*^−1^
ω	frac. of treated with high awareness	0.4 (0,1)	*year*^−1^
π	active case-finding rate	5 (0,30)	*year*^−1^

*See Okuonghae and Omosigho (2011) (and the references therein) for sources of the parameter values*.

From the expressions in the limits of R_*T*_ in Equation (19), it is seen that near total eradication of tuberculosis is achievable. One strategy is to effectively combine making tuberculosis medical tests and treatment free, having high awareness rates (for both susceptible and latently infected persons), continuous enlightenment programmes to reduce the likelihood of loss of awareness (or forgetfulness), high reportage of chronic cough (to improve the detection of likely TB cases) and effective and high treatment rate (i.e., ν → 1, α_1_ → ∞(ψ → ∞), θ_1_ → 0(θ_2_ → 0), α_2_ → ∞, *r* → ∞). It is remarkable that a similar conclusion is reached when a control strategy concentrates on high chronic cough identification rate, making tuberculosis tests and treatment free, high active case-finding rate and a high and effective treatment rate (i.e., ν → 1, α_2_ → ∞, *r* → ∞, π → ∞).

Of course, the above mentioned control strategies could be cost prohibitive and may seem unrealistic, especially in developing countries. However, the alternative strategies that can be gleaned from the expressions in Equations (17) and (18) can be applied to the target community, in reducing the severity of tuberculosis in the community; note that, for example, the limit of R_*T*_ in Equation (17) does not necessarily involve having a high treatment rate especially when such treatment level is lacking in the community.

Computing the partial derivatives of R_*T*_ with respect to the key parameters (*r*_2_, α_2_, α_1_, θ_1_, θ_2_, ψ, π and ν) further reveals the effect of these parameters on tuberculosis control in the population. The derivatives are given in Appendix B.

Clearly, it follows from Equation (A3) (Appendix B) that $\frac{\partial R_{T}}{\partial r}<0$, unconditionally. Therefore, effective treatment rates of tuberculosis will have a positive impact in reducing the disease burden in the population. This result is stated in the following lemma

Lemma 2.2. *Effective treatment will have a positive impact on the TB burden in a community by reducing the incidence of the disease in the population regardless of the values of the other parameters in the expression for the effective reproduction number*.

Also, from Appendix B i.e., Equations (A4), (A5), we see that $\frac{\partial R_{T}}{\partial \alpha_{1}}>0$ $\left(\frac{\partial R_{T}}{\partial \theta_{1}}<0\right)$ if

(20) $$\sigma >\sigma^{*}=\frac{k_{1}k_{2}+k_{1}\theta_{2}+k_{2}\psi +\mu [k_{1}+k_{2}p_{1}+p_{1}(\mu +\theta_{2}+\psi )]}{k_{1}k_{2}+k_{1}\theta_{2}+k_{2}\psi +\mu [k_{1}p_{2}+k_{2}+p_{2}(\mu +\theta_{2}+\psi )]}.$$

However, $\frac{\partial R_{T}}{\partial \alpha_{1}}<0$ $\left(\frac{\partial R_{T}}{\partial \theta_{1}}>0\right)$ if σ < σ^*^ and $\frac{\partial R_{T}}{\partial \alpha_{1}}=0$ $\left(\frac{\partial R_{T}}{\partial \theta_{1}}=0\right)$ when σ = σ^*^. This implies that a high awareness level by some of the susceptible individuals will have a positive impact on the TB burden in the population if σ < σ^*^ i.e., the likelihood of such susceptible individuals getting infected with TB should be less than the computed σ^*^. The strategy of using awareness (and its efficiency on TB control) to combat the disease will fail to reduce the burden of tuberculosis in a community if σ = σ^*^, and, in fact, will have a detrimental impact on the community, by increasing the value of R_*T*_, if σ > σ^*^. This result is reversed if there is a loss of awareness over time by susceptible individuals who previously had a high level of awareness (forgetfulness). Hence, such loss of awareness will bring about a reduction in the value of the effective reproduction number if the likelihood of infection of these susceptible (with previously high level of awareness) is greater than the computed σ^*^, it will have no impact if σ = σ^*^ and will have a detrimental effect on the community if σ < σ^*^. The result is summarized thus:

Lemma 2.3. *A high awareness level for susceptible individuals will have a positive impact on the reduction of the TB burden in a community if* σ < σ^*^, *no impact if* σ = σ^*^ *and a detrimental impact if* σ > σ^*^. *The result is reversed for the rate of loss of awareness by these susceptibles: a positive impact if* σ > σ^*^, *no impact when* σ = σ^*^ *and a detrimental impact if* σ < σ^*^.

This result highlights the need for sustaining the tuberculosis awareness programmes so that forgetfulness could be minimized and the power of the knowledge of TB could help in reducing the likelihood of new tuberculosis infections in the population.

A very close look at the expression for σ^*^, giving in Equation (20), shows the significance of the fraction of fast progressions in the system i.e., *p*_1_ and *p*_2_. Clearly, if *p*_1_ = *p*_2_ = 1, then σ^*^ = 1 (which is also the case when *p*_1_ = *p*_2_ = *p* and *k*_1_ = *k*_2_ = *k*); recall that one major assumption in the model (3) is that, due to the effectiveness of awareness, σ ≤ 1. Therefore, we conjecture that these fractions of fast progressions, *p*_1_ and *p*_2_ and the rate of endogenous reactivation, *k*_1_ and *k*_2_ are very crucial in determining whether σ^*^ is less than, equal to or greater than one. Hence, the impact of awareness or loss of it over time, on the dynamics of tuberculosis, is tied to the critical value of the reduced likelihood of susceptible individuals, with high awareness level, getting infected with TB (σ) and to the fractions of fast TB progressions from new infections and the rates of endogenous reactivations (based on the levels of awareness in the population).

Taking a look at Appendix B, i.e., from Equations (A6), (A7), we observe that $\frac{\partial R_{T}}{\partial \theta_{2}}<0$ $\left(\frac{\partial R_{T}}{\partial \psi }>0\right)$ if

(21) $$k_{1}<k_{2}.$$

This implies that the high awareness level of some latently infected individuals will bring about a reduction in the value of the effective reproduction number (a positive impact) if the progression rate (endogenous reactivation), *k*_1_, of latent individuals with low awareness level, to the active stage of TB is greater than that for latently infected individuals with high awareness level i.e., *k*_1_ > *k*_2_; there will be no impact on TB dynamics if *k*_1_ = *k*_2_ and a detrimental impact when *k*_1_ < *k*_2_. This result is reversed when we investigate the relationship between the effective reproduction number and the rate of loss of awareness by the latently infected individuals who previously had a high awareness level (θ_2_). This leads to the following lemma:

Lemma 2.4. *A high awareness rate for some of the latently infected individuals will have a positive impact on the reduction of the TB burden in a community if *k*_1_ > *k*_2_, no impact if* *k*_1_ = *k*_2_ *and a detrimental impact if* *k*_1_ < *k*_2_. *The result is reversed for the rate of loss of awareness by such latently infected: a positive impact if* *k*_1_ < *k*_2_, *no impact when* *k*_1_ = *k*_2_ *and a detrimental impact if* *k*_1_ > *k*_2_.

This result significantly demonstrates the connection between awareness levels (and loss of awareness over time) of latently infected individuals and their progression rates (endogenous reactivation) to active tuberculosis, on the dynamics of the disease in a community. We therefore conjecture that, from Lemmas 2.3 and 2.4, the effect of the awareness levels (and loss of previously high awareness level) of susceptible and latently infected individuals is tied to some critical parameters: for the former (susceptible), the effect of high awareness level (and lack of it) on the effective reproduction number depends heavily on σ (which further depends on *p*_1_ and *p*_2_, and probably *k*_1_ and *k*_2_) while for the latter (latently infected individuals), the effect of high awareness level (and lack of it) on the effective reproduction number depends only on the progression rates to active TB, *k*_1_ and *k*_2_.

Basically, these Lemmas (2.3 and 2.4) demonstrates that, even when awareness levels wanes probably due to stoppages in enlightenment programmes in several media, then the progression rates (*p*_1_, *p*_2_, *k*_1_ and *k*_2_) plays a significant role in the dynamics of tuberculosis in the population and their effect should be harnessed by health officials for proper control of the disease.

From the analysis and results stated in Lemmas 2.3 and 2.4, it seems that the progression and reactivation rates (*p*_1_, *p*_2_, *k*_1_, and *k*_2_) can vary from individual to individual or from community to community. Generally, tuberculosis is most likely to occur in the first year following infection, with stepwise reduction year on year over the following 5–10 years, by which time incidence approaches that of uninfected contacts (Esmail et al., 2014). Also, reactivation several decades after initial infection occurs, but beyond 10 years, it is difficult to assess how common reactivation is (Esmail et al., 2014). Also, we earlier stated that the period of fast latency can span between 1 and 5 years (Styblo, 1980; Flynn and Chan, 2001; Colijn et al., 2007). Hence, the time line for the risk of disease over time is, generally, not fixed, as this can be determined by several biological and environmental factors, so that we can assume that the parameters that describe disease progressions can vary within some reasonable bounds.

Looking at Appendix B, i.e., Equations (A8), (A9), and (A10), we observe that $\frac{\partial R_{T}}{\partial \pi }<0$, $\frac{\partial R_{T}}{\partial \alpha_{2}}<0$, and $\frac{\partial R_{T}}{\partial \nu }<0$ if

(22) $$d<d^{*}=\frac{(1-\eta )\mu +r}{\eta },\;\eta \neq 0$$

or

(23) $$\eta <\eta^{*}=\frac{\mu +r}{\mu +d}$$

The results in Equations (22) and (23) reveals that the active case-finding strategy, the use of chronic cough as a marker for identifying potential TB cases, together with an efficient cost factor, will bring about a reduction in the value of the effective reproduction number if the disease-induced death rate is less than the computed d^*^ (22) or for the relative infectiousness of individuals with active TB who are detected for immediate treatment to be less than the computed η^*^ (23). The aforementioned parameters will have no impact on TB control if *d* = d^*^ or η = η^*^ but will have a detrimental effect on tuberculosis control and the TB burden in the population if *d* > d^*^ or η > η^*^. We can state the following lemma:

Lemma 2.5. *A high active case-finding and chronic cough identification rates with a low cost factor will have a positive impact on the reduction of the TB burden in a community if* *d* < d^*^(η < η^*^), *no impact if* *d* = d^*^(η = η^*^) *and a detrimental impact if* *d* > d^*^(η > η^*^).

Recall that we assume that the modification parameter η ≤ 1. Therefore, we expect that η^*^ ≤ 1, which implies that *r* ≤ *d*. Hence, if the disease-induced death rate is greater than the treatment rate, then the active case-finding strategy, coupled with the effective use of chronic cough identification and an effective cost factor will have a positive impact on the dynamics of tuberculosis and bring down the TB burden in the population.

From the analysis carried out on R_*T*_, one observes that taking treatment alone without considering the effect of other parameters on the dynamics of tuberculosis, may not be enough in reducing the TB burden in the population. Clearly, a critical combination of two or more parameters from the set of key parameters, notably ν, α_1_, α_2_ and others like θ_1_, π, ψ and θ_2_, can significantly affect the value of the effective reproduction number. These parameters also affect the number of detected cases (which improves the case detection and notification rates) and, with effective treatment, will reduce the tuberculosis burden in the population.

#### 2.2.5. Backward bifurcation analysis: special case

The phenomenon of backward bifurcation, which has been observed in several disease transmission models (see, e.g., Hadeler and van den Driessche, 1997; Castillo-Chavez and Song, 2004), is typically characterized by the coexistence of a stable DFE and a stable endemic equilibrium when the associated reproduction number of the model is less than unity. The public health implication of the backward bifurcation phenomenon of model (3) is that the classical epidemiological requirement of having the reproduction number (R_*T*_) be less than one, although necessary, is no longer sufficient for effective control of the disease in the population.

We can determine the possibility of the existence of a backward bifurcation in model (3); this is possible when we check for the existence of multiple endemic equilibria when the reproduction number of model (3) is less than one.

Consider the case when there is no treatment in the model (3) (this is fitting in communities where treatment facilities are not available or not enough to cater for the affected individuals) and insignificant disease-induced death rate i.e., ϵ = *r* = *d* = 0. It should be noted that setting *d* = 0 in (3) gives $N\left(t\right)\to \frac{\Lambda }{\mu }$ as *t* → ∞. Let $\hat{\beta }=\frac{\mu \beta }{\Lambda }$ so that the force of infection now becomes

(24) $$\lambda =\hat{\beta }(I+\eta J).$$

Also, let $\hat{R_{T}}$ be the effective reproduction number of model (3) with ϵ = *r* = *d* = 0.

To find the conditions for the existence of the endemic equilibrium for the model (3) with ϵ = *r* = *d* = 0, denoted by $\xi_{2}=\left(S_{1}^{**},S_{2}^{**},E_{1}^{**},E_{2}^{**},I^{**},J^{**}\right)$, the Equation in (3), with ϵ = *r* = *d* = 0, are solved in terms of the force of infection, at steady state [using Equation (24)] (where the force of infection at steady state will be denoted by λ^*e*^), and this must satisfy the following polynomial:

(25) $$f(\lambda^{e})=A_{1}\lambda^{e}{}^{4}+A_{2}\lambda^{e}{}^{3}+A_{3}\lambda^{e}{}^{2}+A_{4}\lambda^{e}+A_{5}=0$$

where the coefficients, *A*_1_, …, *A*_5_ and the endemic equilibrium, ξ_2_, are given in Appendix C.

The components of the endemic equilibrium, given in Equation (A11) (Appendix C), are then obtained by solving for λ^*e*^ from the quartic (25), and substituting the positive values of λ^*e*^ into the expressions of the endemic steady states given in Equation (A11). Furthermore, it follows that the coefficient *A*_1_, of the quartic (25), is always positive, and *A*_5_ is positive (negative) if ${\hat{R}}_{T}$ is less (greater) than one. The following can be deduced:

Theorem 2.3. *The treatment-free model of* (3), *with d* = 0, *has*

*i no endemic equilibrium if b*_1_ = 0 *and* ${\hat{R}}_{T}<1$ *(absence of exogenous re-infection from the E*_1_ *class)*.
*ii has four or two endemic equilibria if A*_2_ < 0, *A*_3_ > 0, *A*_4_ < 0 *and* ${\hat{R}}_{T}<1$.
*iii has two endemic equilibria if A*_3_ *is of the same sign as A*_2_ or *A*_4_ *and* ${\hat{R}}_{T}<1$.
*iv has two endemic equilibria if A*_2_ > 0, *A*_3_ < 0, *A*_4_ > 0 *and* ${\hat{R}}_{T}<1$.
*v no endemic equilibrium otherwise when* ${\hat{R}}_{T}<1$.
*vi a unique endemic equilibrium when* ${\hat{R}}_{T}>1$.

Items (ii)—(iv) of Theorem 2.3 suggests the possibility of backward bifurcation in the treatment-free model, with *d* = 0. Determining specific parameters that could be the cause of the phenomenon, for the system (3), is quite challenging due to the number of parameters in the model and the degree of the polynomial (25). However, since it has been established that exogenous re-infection can trigger the backward bifurcation phenomenon in the dynamics of tuberculosis (Hadeler and van den Driessche, 1997; Castillo-Chavez and Song, 2004; Okuonghae and Omosigho, 2011), it will be interesting to observe the effects of both exogenous re-infection parameters in the system i.e., *b*_1_ and *b*_2_, since these parameters are based on the levels of awareness of the latently infected individuals.

First of all, it is important to show that in the absence of exogenous re-infection from the latently infected class of individuals with low awareness level, *b*_1_ = 0, the system (3) will not have a backward bifurcation when ${\hat{R}}_{T}<1$. Clearly, setting *b*_1_ = 0 in Equation (25), yields the quadratic equation

(26) $$f(\lambda^{e})={\hat{A}}_{3}\lambda^{e}{}^{2}+{\hat{A}}_{4}\lambda^{e}+A_{5}=0$$

where Â_3_ and Â_4_ are now evaluated from *A*_3_ and *A*_4_, respectively, with *b*_1_ = 0. It is easy to see that Â_3_ > 0 and *A*_5_ > 0 (the latter when ${\hat{R}}_{T}<1$). We want to show that, since σ ≤ 1, the coefficient Â_4_ > 0, so that the quadratic (26) will not have any positive roots, which implies that there is no endemic equilibrium when *b*_1_ = 0 in Equation (25). With *b*_1_ = 0 in Equation (25), we see that

$$\begin{array}{l} {\hat{A}}_{4}=\Lambda [(\mu +\pi +\nu \alpha_{2})(\mu +\mu \sigma +\sigma \alpha_{1}+\theta_{1})((\mu +k_{1})\\ \;(\mu +k_{2}+\theta_{2})+(\mu +k_{2})\psi )]-\Lambda [\beta (\mu +\eta (\pi +\nu \alpha_{2}))\\ \;\sigma ((k_{1}+\mu p_{1})(\mu +k_{2}+\theta_{2})+(k_{2}+\mu p_{1})\psi )] \end{array}$$

Since σ ≤ 1, it then implies that,

(27) $$\begin{array}{l} {\hat{A}}_{4}\geq \Lambda [(\mu +\pi +\nu \alpha_{2})(\mu +\mu \sigma +\sigma \alpha_{1}+\theta_{1})((\mu +k_{1})\\ \;(\mu +k_{2}+\theta_{2})+(\mu +k_{2})\psi_{1})]-\Lambda [\beta (\mu +\eta (\pi +\nu \alpha_{2}))\\ \;((k_{1}+\mu p_{1})(\mu +k_{2}+\theta_{2})+(k_{2}+\mu p_{1})\psi_{1})]. \end{array}$$

From ${\hat{R}}_{T}<1$, it follows that

$$\hat{\beta }\leq \frac{G_{4}}{\left(\mu +\eta \pi +\eta \nu \alpha_{2})(G_{1}+G_{2}+G_{3}\right)},$$

where $\hat{\beta }$ is now evaluated with *r* = *d* = 0. Substituting the expression for $\hat{\beta }$ into Equation (27), we see that

(28) $$\begin{array}{l} {\hat{A}}_{4}\geq \Lambda [(\mu +\pi +\nu \alpha_{2})(\mu +\mu \sigma +\sigma \alpha_{1}+\theta_{1})((\mu +k_{1})\\ \;(\mu +k_{2}+\theta_{2})+(\mu +k_{2})\psi_{1})]\\ \;-\Lambda [(\frac{G_{4}}{\left(\mu +\eta \pi +r_{2}+\eta \nu \alpha_{2})(G_{1}+G_{2}+G_{3}\right)})(\mu +\eta \\ \;(\pi +\nu \alpha_{2}))((k_{1}+\mu p_{1})(\mu +k_{2}+\theta_{2})+(k_{2}+\mu p_{1})\psi_{1})]. \end{array}$$

Simplifying this further, we have that

$$\begin{array}{l} {\hat{A}}_{4}\geq \Lambda [(\mu +\pi +\nu \alpha_{2})[((\mu +k_{1})(\mu +k_{2}+\theta_{2})+(\mu +k_{2})\psi )\\ \;\;\;\;\;\;\;\;\;\;\;(k_{1}(\mu^{3}+k_{2}{\left(\mu +\alpha_{1}+\theta_{1}\right)}^{2}+\mu (2\mu +\alpha_{1}+\theta_{1})(p_{2}\alpha_{1}+\theta_{1})\\ \;\;\;\;\;\;\;\;\;\;+{\left(\mu +\alpha_{1}+\theta_{1}\right)}^{2}\theta_{2})+\mu \alpha_{1}p_{2}(2\mu +\alpha_{1}+\theta_{1})(\mu +\theta_{2}+\psi )\\ \;\;\;\;\;\;\;\;\;\;+\mu p_{1}(\mu^{2}+\theta_{1}(2\mu +\alpha_{1}+\theta_{1}))(\mu +k_{2}+\theta_{2}+\psi )\\ \;\;\;\;\;\;\;\;\;\;+k_{2}(\mu \alpha_{1}(2\mu +\alpha_{1}+\theta_{1})+{\left(\mu +\alpha_{1}+\theta_{1}\right)}^{2}\psi ))]>0, \end{array}$$

for σ ≤ 1. Therefore, the polynomial (26) will not have a positive root, hence no endemic equilibrium, when *b*_1_ = 0 and ${\hat{R}}_{T}<1$, ruling out the possibility of a backward bifurcation, for this case.

However, when *b*_2_ = 0 and *b*_1_ ≠ 0 (in this case, there is no exogenous re-infection of latently infected individuals with high awareness level but there is exogenous re-infection in the *E*_1_ class i.e., latently infected individuals with low level of awareness), the quartic (25) now reduces to a cubic equation

(29) $$f(\lambda^{e})={Ã}_{2}\lambda^{e}{}^{3}+{Ã}_{3}\lambda^{e}{}^{2}+{Ã}_{4}\lambda^{e}+A_{5}=0$$

where $\tilde{A_{2}},\tilde{A_{3}}$ and $\tilde{A_{4}}$ are obtained from evaluating the corresponding coefficients of (25) with *b*_2_ = 0.

Following the analysis above, it is easy to show that the polynomial (29) can have more than one positive root (hence more than one endemic equilibria) when $\hat{R_{T}}<1$, suggesting the presence of a backward bifurcation for the case *b*_2_ = 0 and *b*_1_ ≠ 0. We can now summarise the above discussions thus:

Lemma 2.6. *The treatment-free model of* (3) with *d* = 0 *can undergo the backward bifurcation phenomenon when*

*i both exogenous re-infection parameters, b*_1_ *and b*_2_, *are present in the system i.e., b*_1_ ≠ 0 *and b*_2_ ≠ 0.
*ii only the exogenous re-infection parameter b*_1_ *is present in the system i.e., b*_1_ ≠ 0 *and* *b*_2_ = 0

*The treatment-free model of (3) with d* = 0 *cannot undergo the backward bifurcation phenomenon when*

*i both exogenous re-infection parameters, b*_1_ *and b*_2_, *are absent in the system i.e., b*_1_ = 0 *and* *b*_2_ = 0.
*ii the exogenous re-infection parameter b*_1_ *is absent in the system (even when b*_2_ ≠ 0) *i.e., b*_1_ = 0 *and* *b*_2_ ≠ 0.

**Figure 5:**
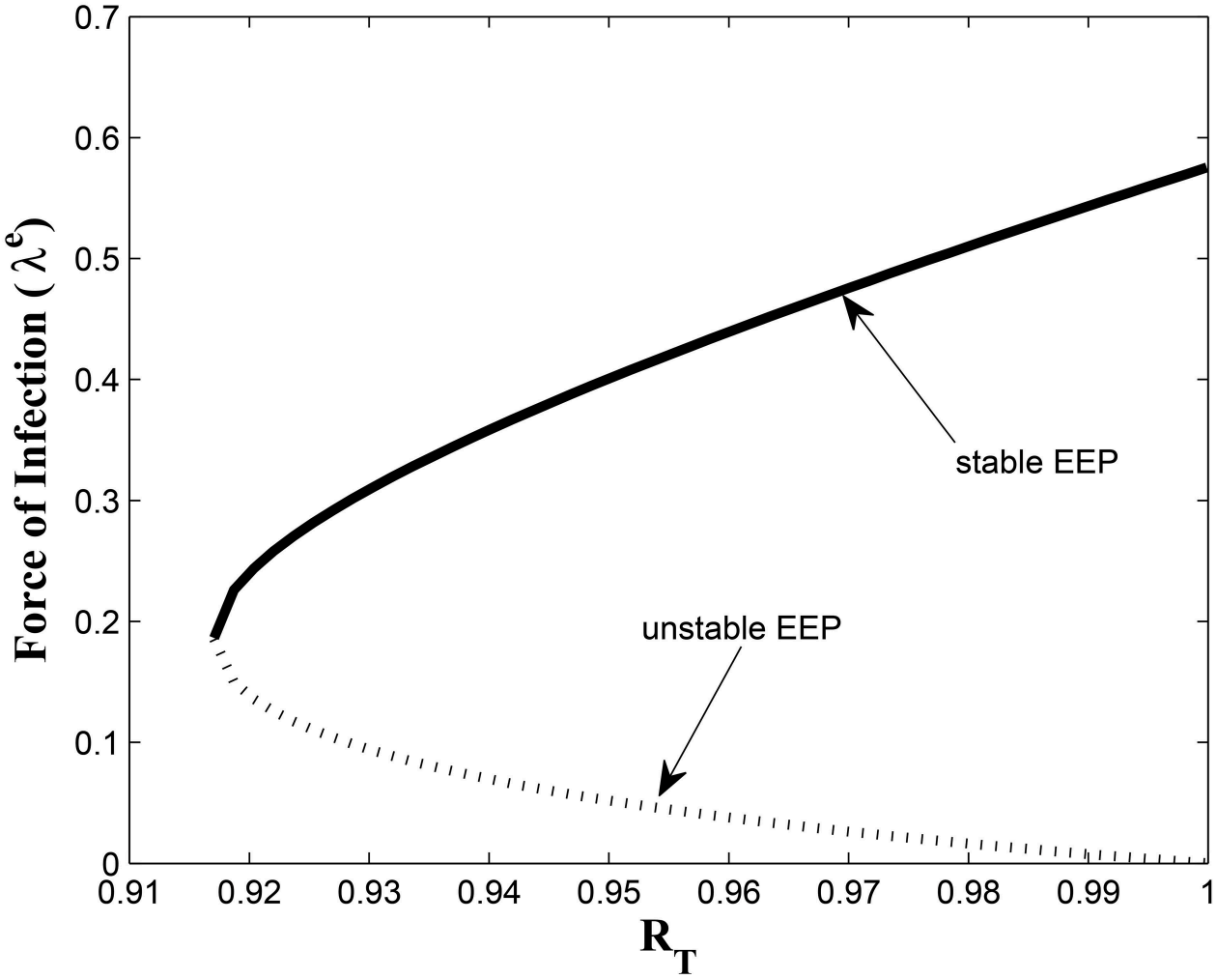
**Backward bifurcation when *b*_1_ ≠ 0 and *b*_2_ ≠ 0**.

**Figure 6:**
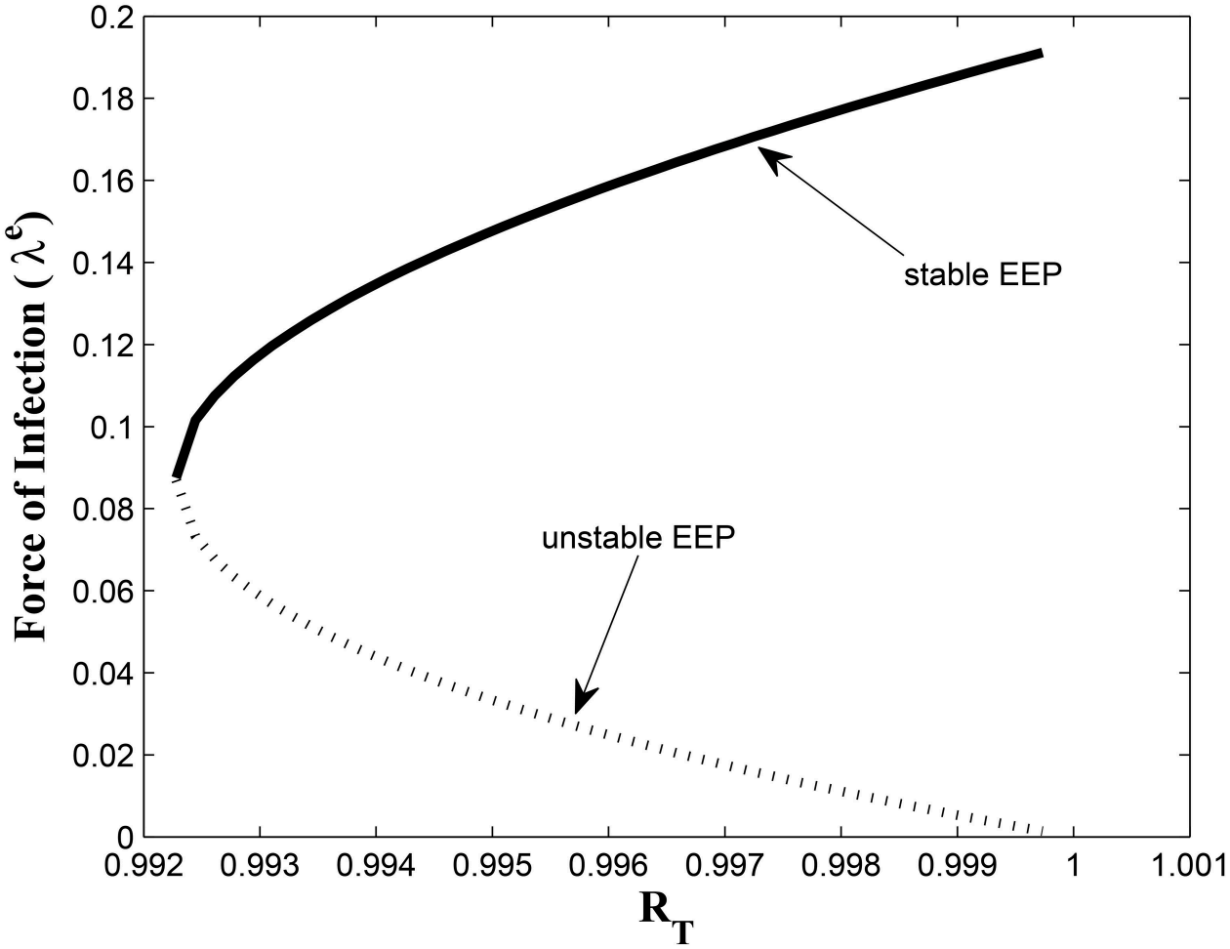
**Backward bifurcation when *b*_1_ ≠ 0 and *b*_2_ = 0**.

This study has, to the best of our knowledge, showed for the first time the critical relationship between TB awareness, heterogeneity in exogenous re-infection (by virtue of the level of awareness of the latently infected individuals) and the tuberculosis burden in a community. We can see, from Lemma 2.6, that exogenous re-infection of latently infected individuals with low awareness level will more negatively impact on TB control than incidences of exogenous re-infection in the latently infected group with high awareness level, due to the influence of the former in allowing for the existence of a backward bifurcation in the system, when the associated reproduction number is less than one. This can be seen from the backward bifurcation diagrams in Figures 5, 6; clearly, in Figure 5, the backward bifurcation range is larger than what we have in Figure 6. Interestingly, when we set *b*_1_ = 0 (the exogenous re-infection parameter for the latently infected individuals with low awareness level), there is no backward bifurcation in the system when the associated reproduction number is less than unity, regardless of the value of *b*_2_ (the exogenous re-infection parameter for the latently infected individuals with high awareness level).

Characterizing the backward bifurcation phenomenon for the complete model (3) is not trivial. However, using the Center Manifold Theorem (Carr, 1981; Castillo-Chavez and Song, 2004; Okuonghae and Omosigho, 2011), we can show the condition required for the system (3) to undergo the backward bifurcation phenomenon when R_*T*_ < 1. We claim the following:

Theorem 2.4. *The model* (3) *exhibits backward bifurcation at* R_*T*_ = 1 *whenever the bifurcation coefficients, denoted by a and b (and given by* (A15) *and* (A16) *in Appendix D, respectively), are positive. However, if the coefficient *a* is negative, then the system* (3) *will not undergo a backward bifurcation at* R_*T*_ = 0.

See Appendix D for proof of Theorem 2.4.

Of course determining, by inspection, which parameter(s) could cause the phenomenon of backward bifurcation in the model Equation (3) [by checking for specific parameter(s) that will make the bifurcation coefficients *a*, given in Equation (A15), to be negative and *b*, given in Equation (A16), to be positive] is non-trivial.

However, we conjecture that, in addition the exogenous re-infection parameters, *b*_1_ and *b*_2_, there could be other parameters that could determine whether the system (3) can undergo a backward bifurcation at R_*T*_ = 1. These parameters, we believe, are liked to the awareness parameters (i.e., α_1_, ψ, θ_1_, and θ_2_) and the fractions of fast progressions, *p*_1_ and *p*_2_. Their overall effect on the backward bifurcation phenomenon for the model (3) is left for future work.

## 3. Results

Model (3) is now numerically simulated with the parameter estimates in Table 1 to gain insight into some of its quantitative features. The system of equations in model (3) was solved numerically using MATLAB; we used the ode45 solver, which is based on the Runge-Kutta method. The parameter values used were based on what exists in literature such as the authors previous works (Okuonghae and Omosigho, 2011) and other references cited in Okuonghae and Omosigho (2011). Few parameter values were assumed, as seen (Okuonghae and Omosigho, 2011) and other relevant literature cited in Okuonghae and Omosigho (2011). The numerical simulations are performed to illustrate various dynamical regimen characteristics by varying some of the key parameters in the model. The effects of varying some of the key parameters on the infected classes are presented and we will examine practicable preventive measures characterized by these variations. Parameter values that are different from those stated in Table 1 are shown in the caption of the respective figure.

**Figure 7:**
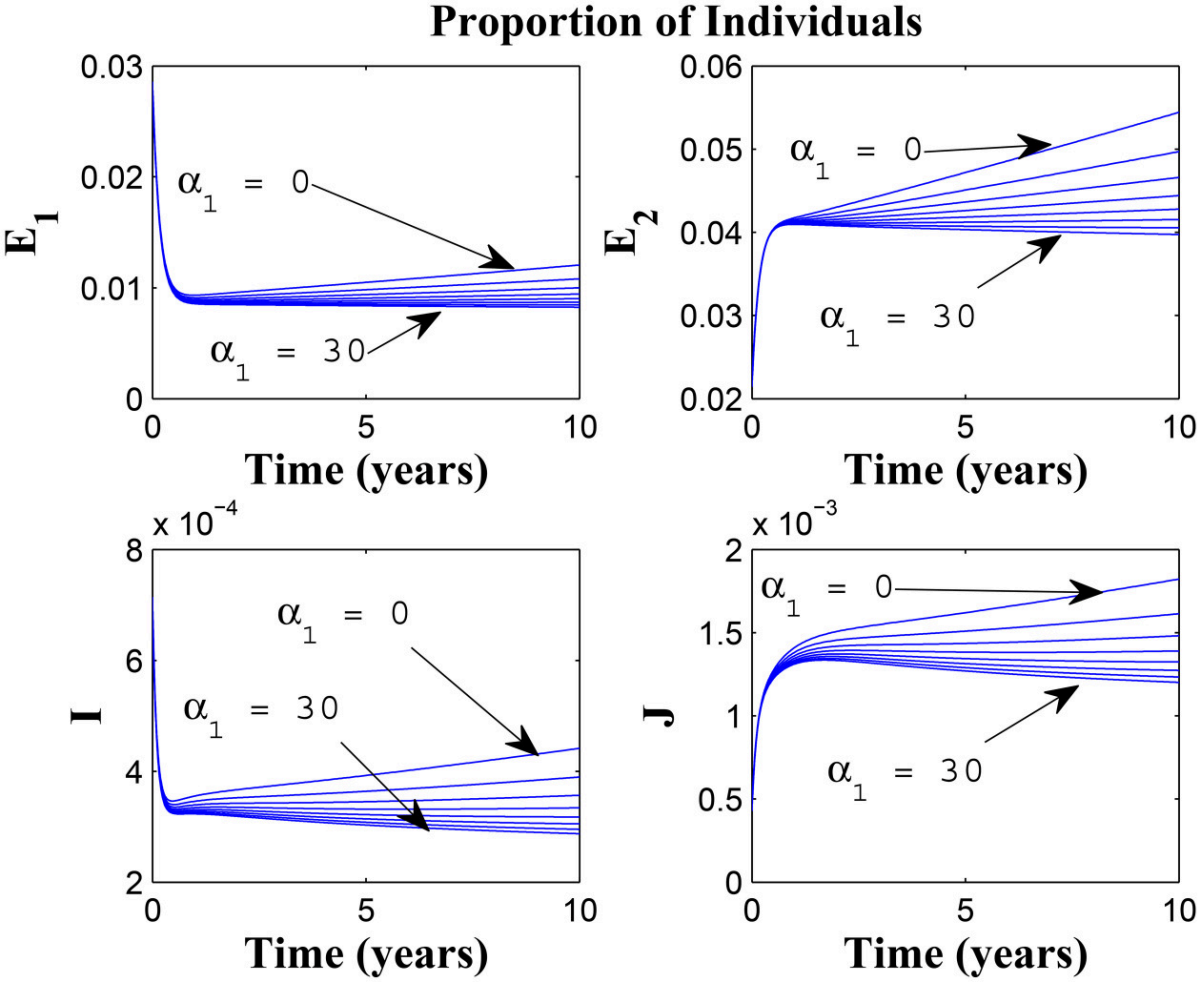
**Simulations of model (3) showing the number of infected individuals**. Here, the awareness rate for the susceptible individuals (α_1_) is varied from 0 to 30 with θ_1_ = 20, θ_2_ = 1and π = 5.

**Figure 8:**
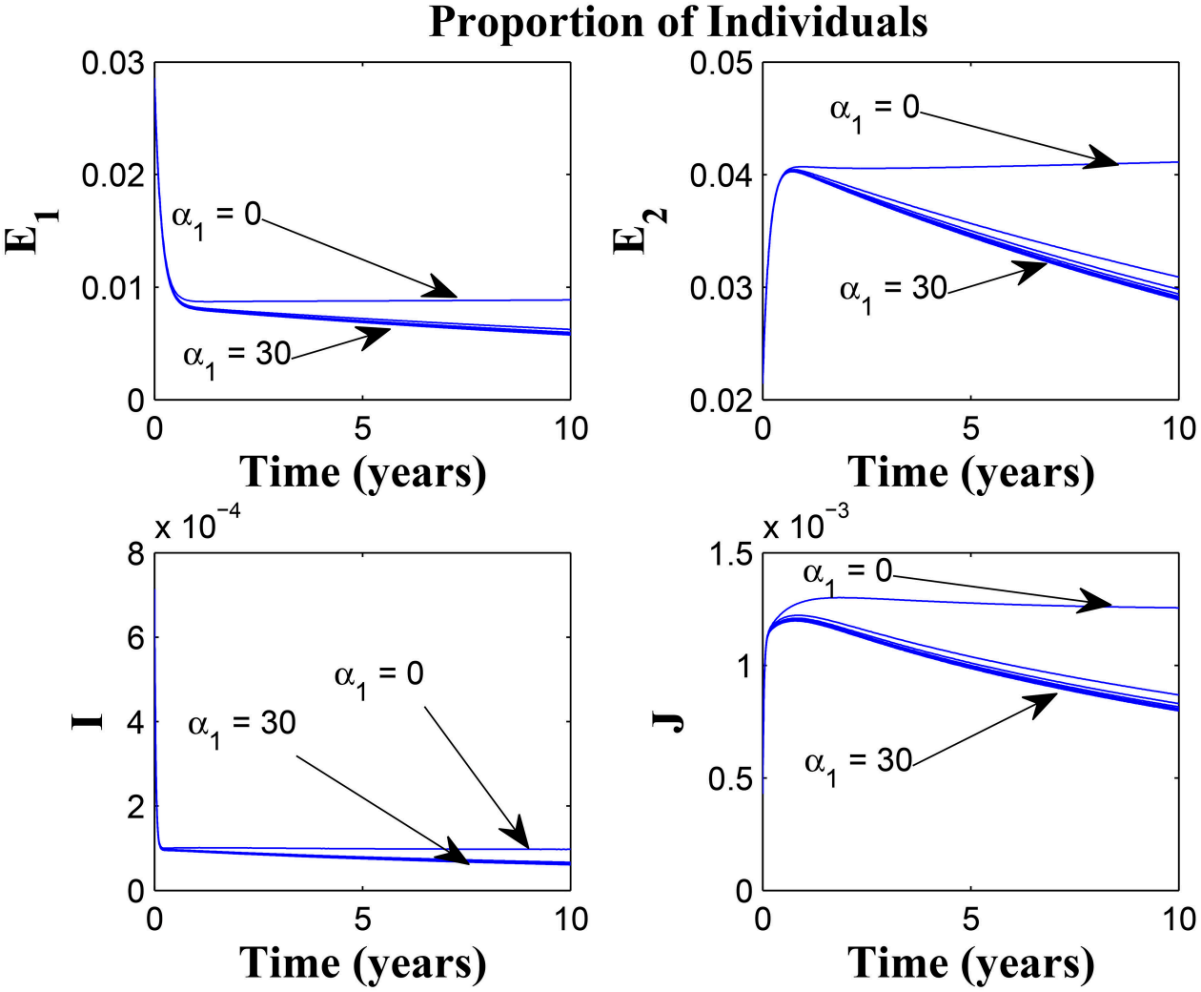
**Simulations of model (3) showing the number of infected individuals**. Here, the awareness rate for the susceptible individuals (α_1_) is varied from 0 to 30 with θ_1_ = 20, θ_2_ = 1 and π = 30.

Figures 7, 8 shows the effect of varying the awareness rate of the susceptible population on the infected classes, vis à viz increasing the active case-finding rate. Clearly, we observe that, even with loss of awareness on the part of susceptible individuals (who previously had a high awareness rate), increasing the active case-finding rate affected (positively) the dynamics of the system with a reduction in the proportion of individuals in the infected classes, and not just the infectious class (*I*), only.

**Figure 9:**
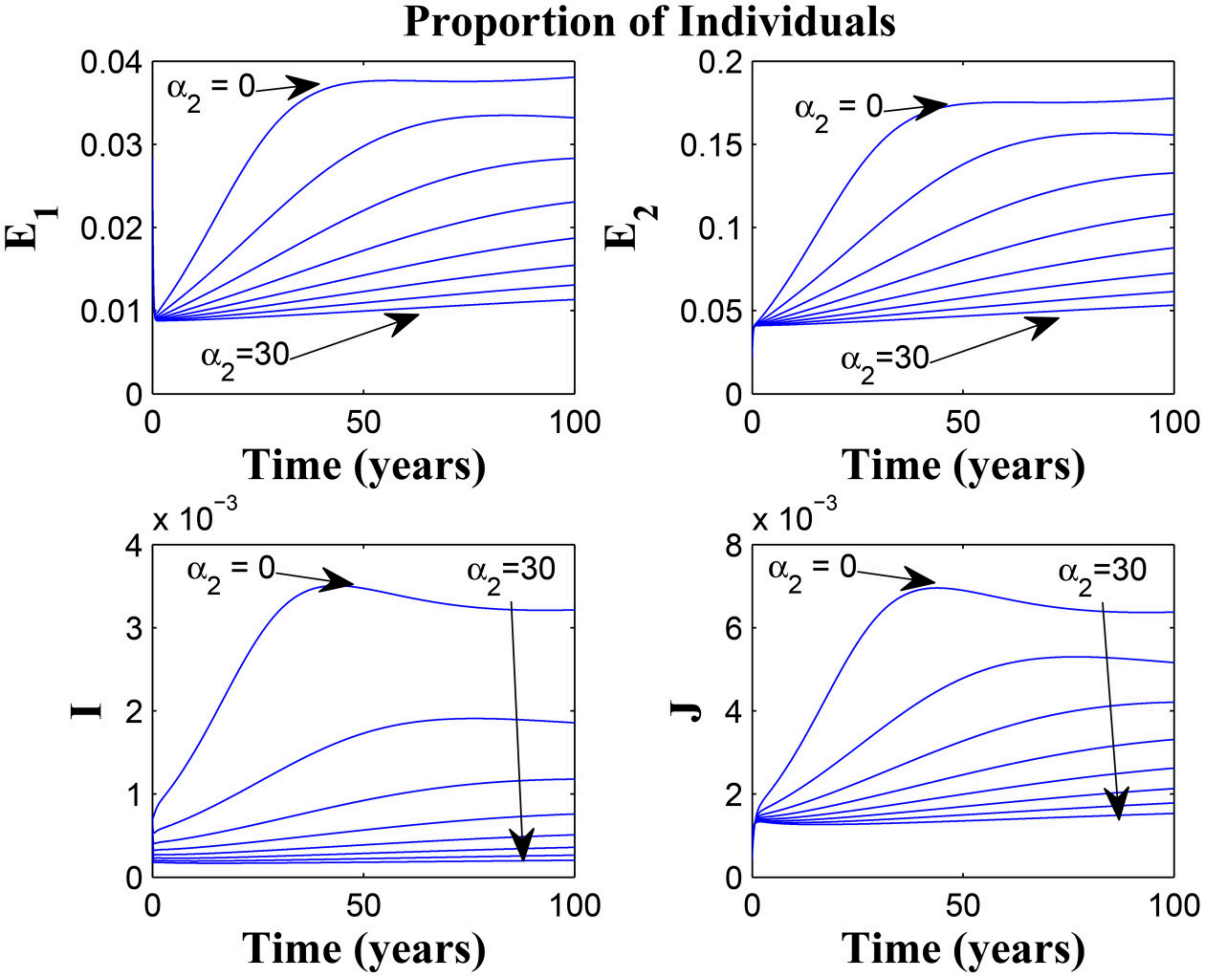
**Simulations of model (3) showing the number of infected individuals**. Here, the cough identification rate (α_2_) is varied from 0 to 30 with θ_1_ = 20 and θ_2_ = 1.

**Figure 10:**
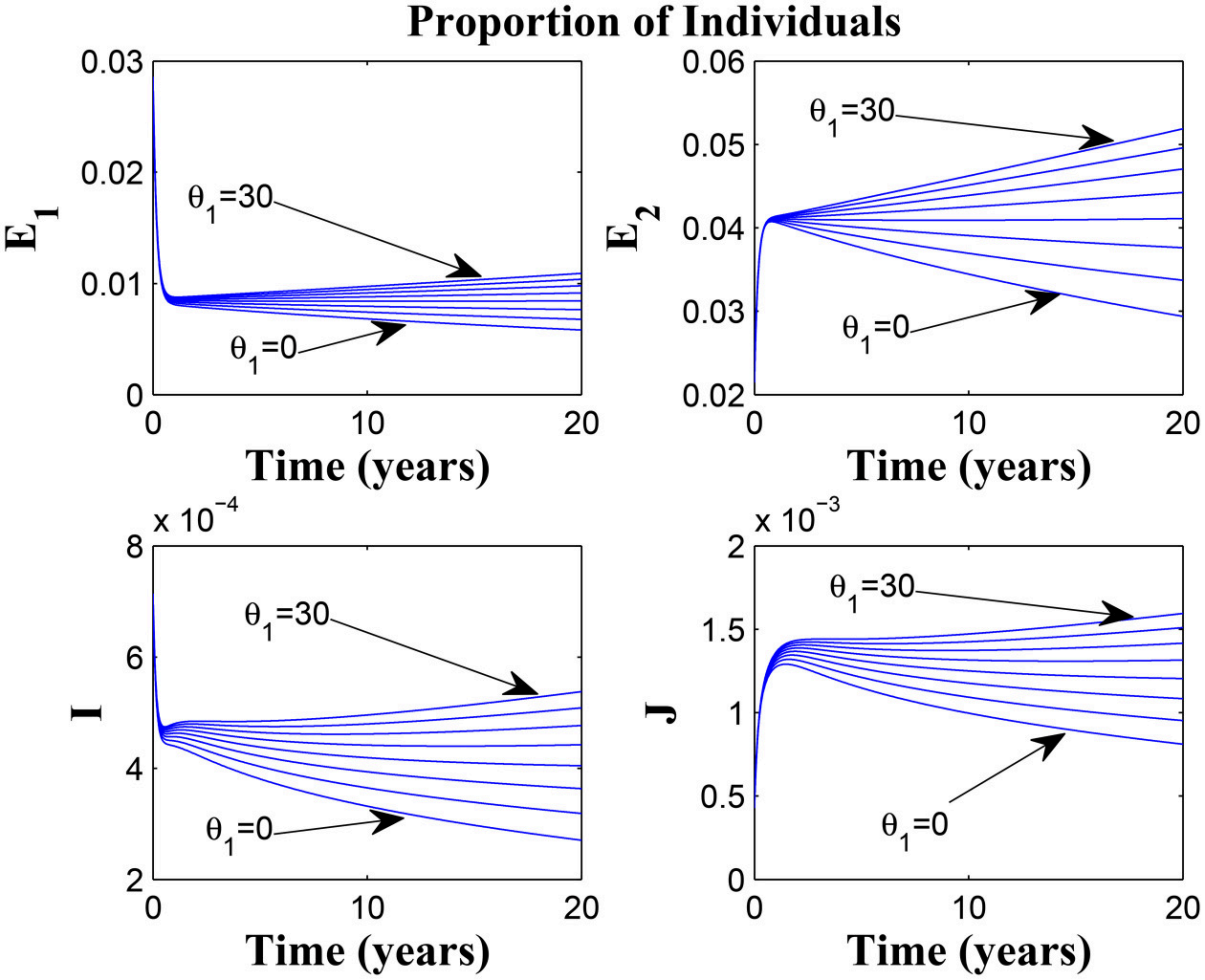
**Simulations of model (3) showing the number of infected individuals**. Here, the rate at which susceptibles lose awareness (θ_1_) is varied from 0 to 30 with α_1_ = 30 and α_2_ = 5.

Figure 9 shows that increasing the chronic cough identification rate (and by implication improving the case detection rate) significantly reduced the proportion of infected individuals, even with a “high” loss of awareness in the susceptible group while Figure 10 shows a worse case scenario whereby an increase in loss of awareness in the susceptible group leads to an increase in the proportion of infected individuals.

**Figure 11:**
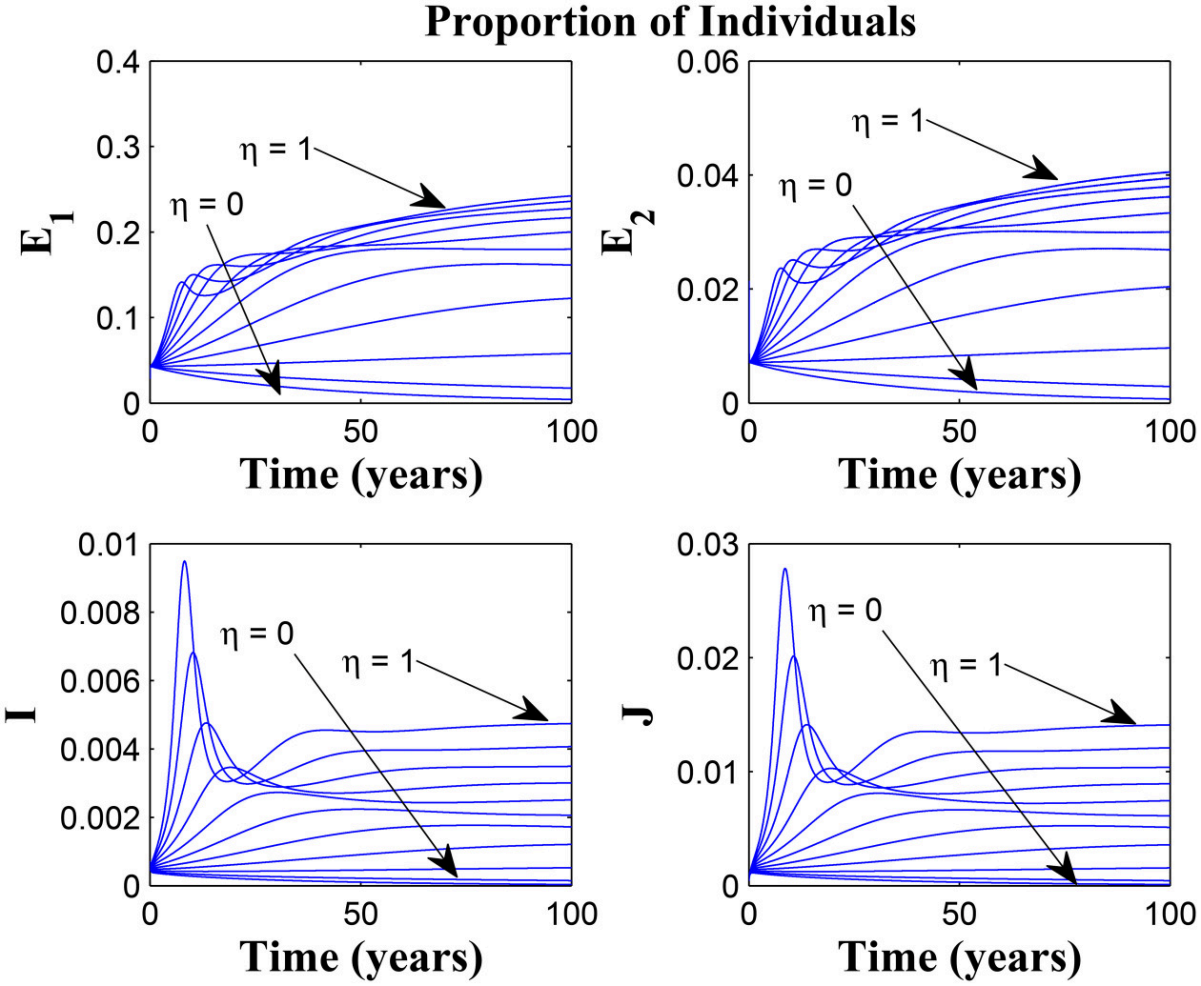
**Simulations of model (3) showing the number of infected individuals**. Here, the disease transmission modification parameter (η) is varied from 0 to 1 with α_1_ = α_2_ = 5, θ_1_ = θ_2_ = 30 and ψ = 5.

**Figure 12:**
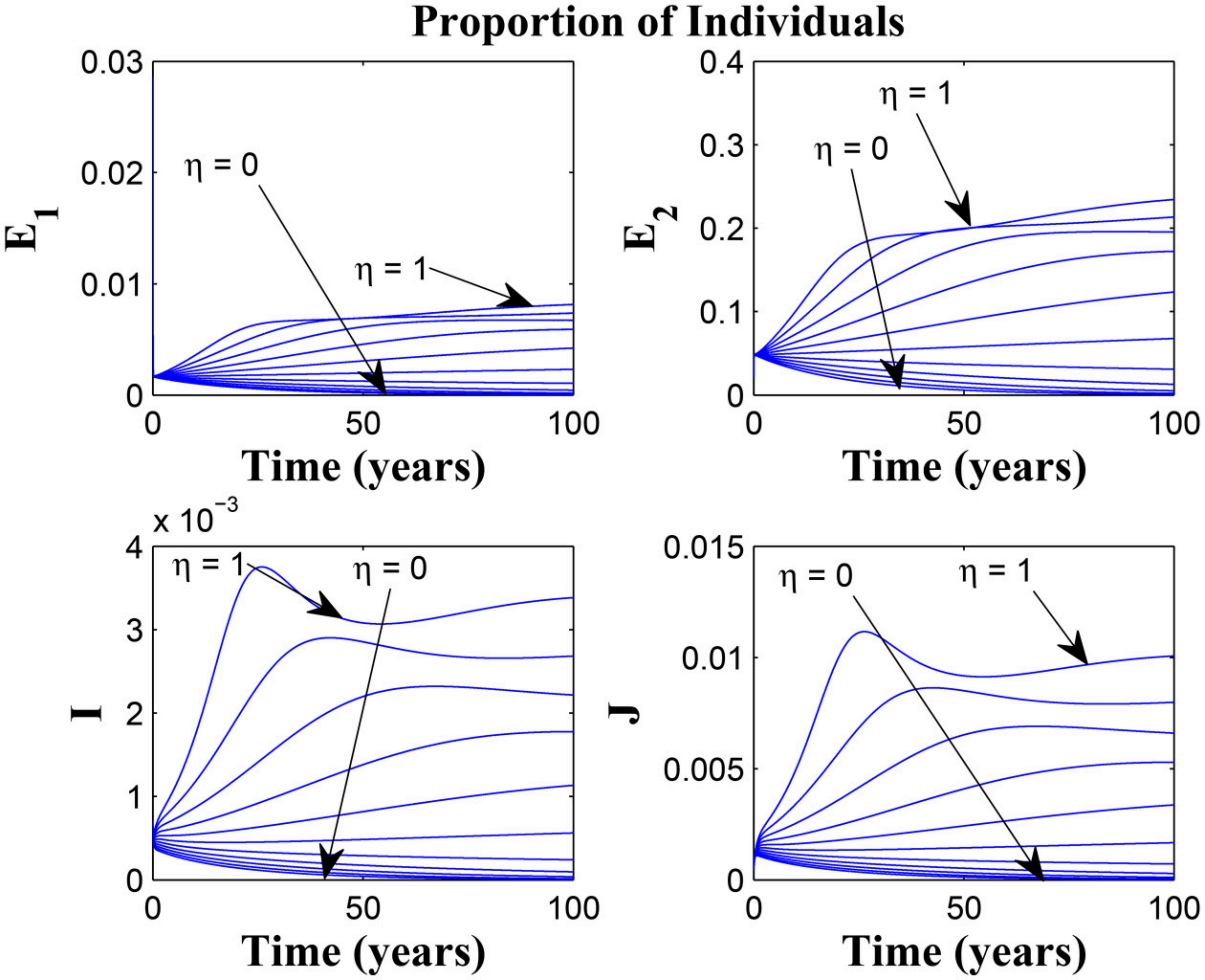
**Simulations of model (3) showing the number of infected individuals**. Here, the disease transmission modification parameter (η) is varied from 0 to 1 with α_1_ = α_2_ = 5, θ_1_ = θ_2_ = 1 and ψ = 30.

Figures 11, 12 shows the proportion of individuals in the infected classes when we vary the reduced likelihood of infection by detected infectious TB cases receiving treatment, η, between 0 and 1. Of course, as expected, reducing the value of η, from 1 to 0, brings down the proportion of infected individuals, albeit at different rates, depending on the values of the awareness parameters for the susceptible and latently infected populations.

**Figure 13:**
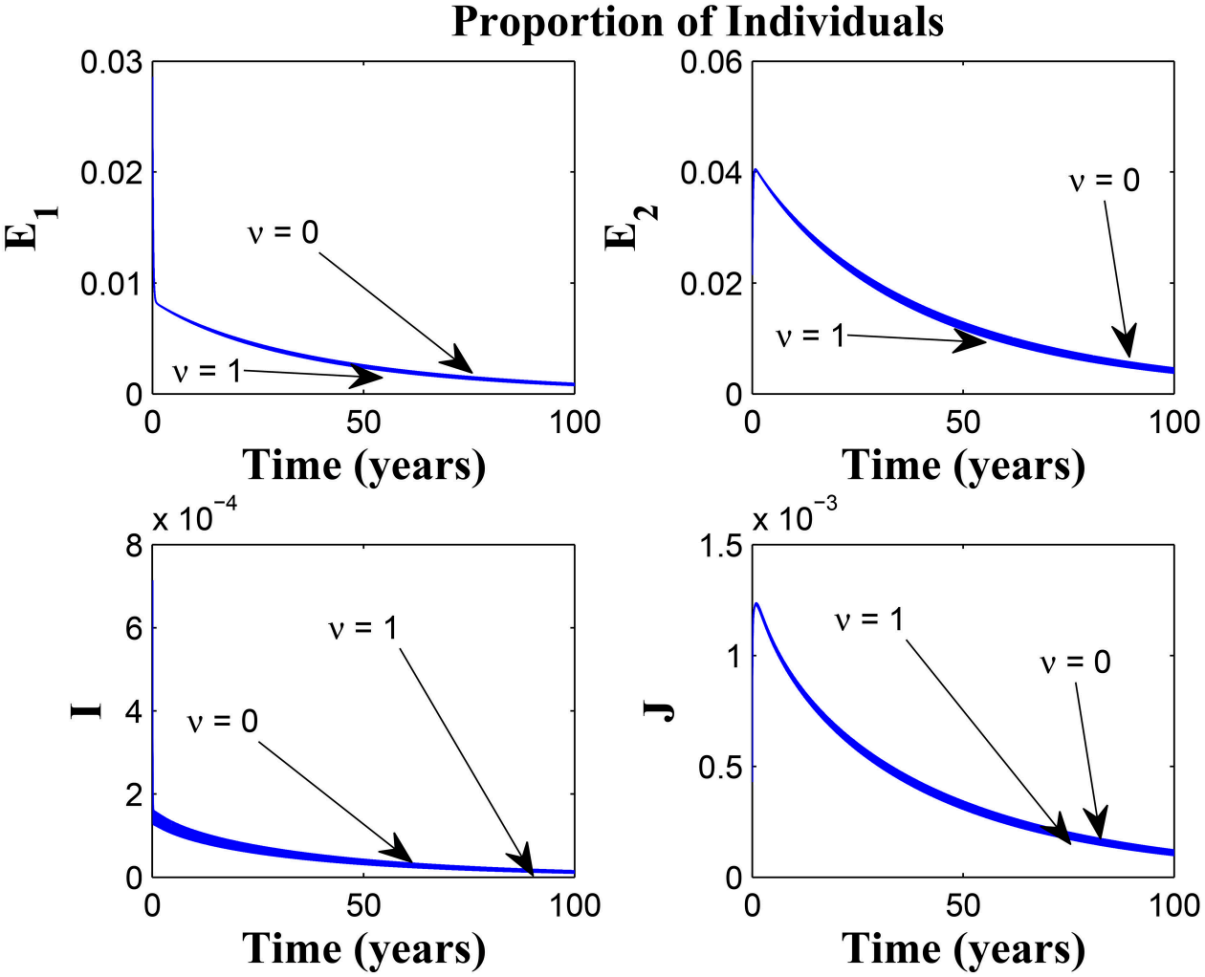
**Simulations of model (3) showing the number of infected individuals**. Here, the cost factor (ν) is varied from 0 to 1 with α_1_ = α_2_ = 5, θ_1_ = θ_2_ = 1, ψ = 30 and π = 20.

Remarkably, Figure 13 shows that there is little influence of the cost factor (ν) on the proportion of infected individuals, in the presence of awareness, chronic cough identification, less loss of awareness and an impressive awareness rate for the latently infected individuals and active case-finding rate.

**Figure 14:**
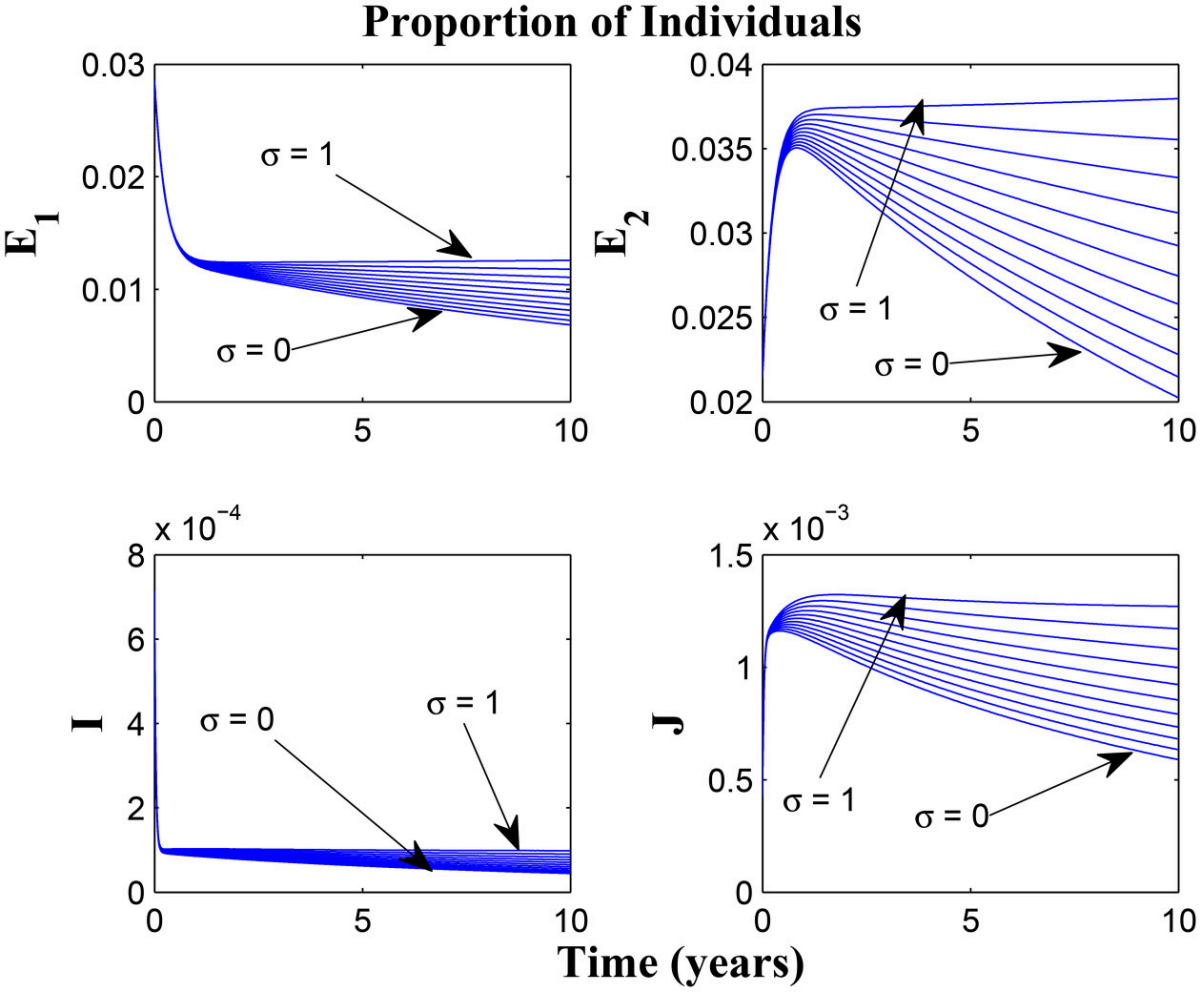
**Simulations of model (3) showing the number of infected individuals**. Here, the reduced likelihood of infection due to awareness (σ) is varied from 0 to 1 with α_1_ = α_2_ = 5, θ_1_ = θ_2_ = 1 and ψ = 30.

**Figure 15:**
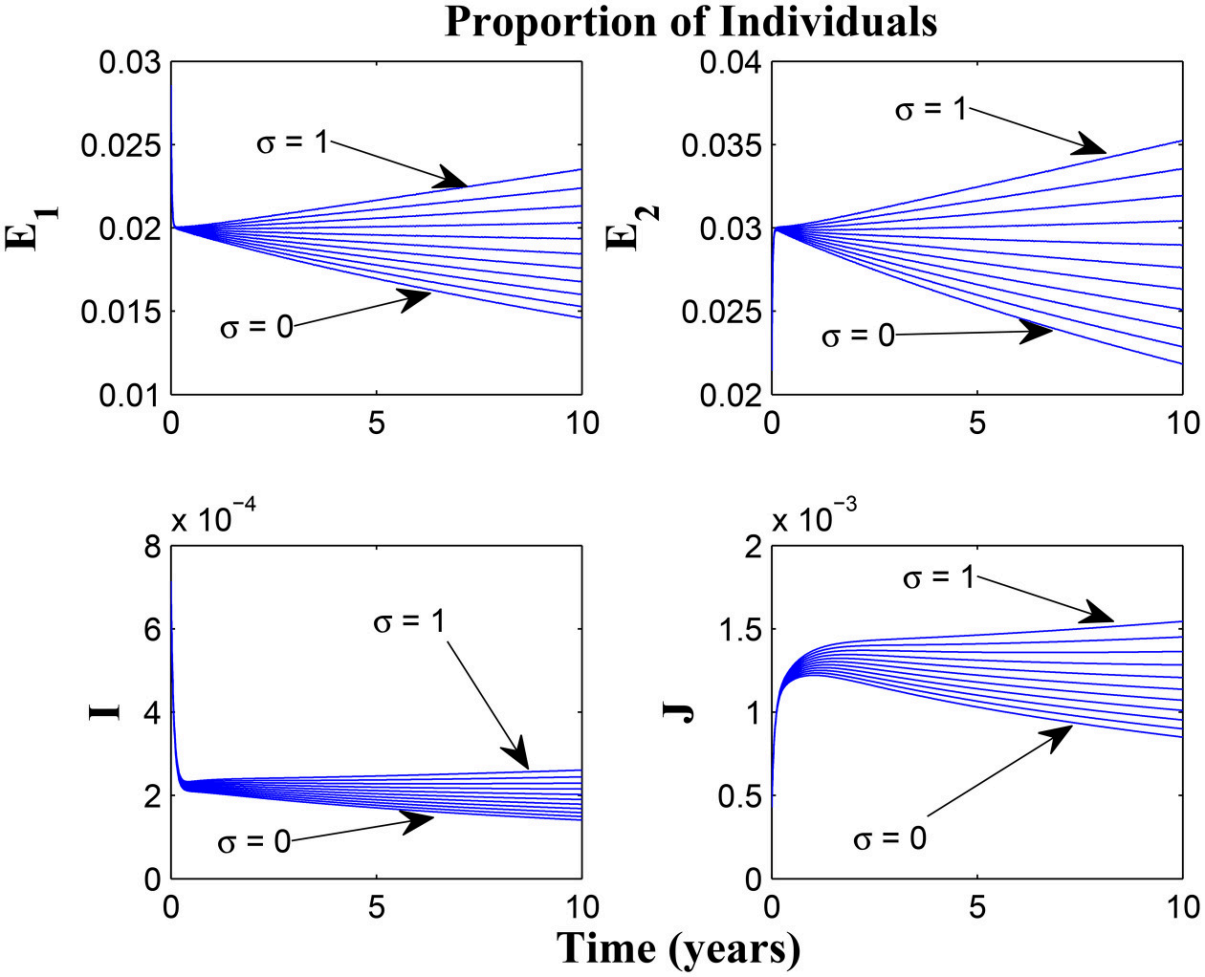
**Simulations of model (3) showing the number of infected individuals**. Here, the reduced likelihood of infection due to awareness (σ) is varied from 0 to 1 with α_1_ = α_2_ = 20, θ_1_ = θ_2_ = 20 and ψ = 30.

Taking a look at Figures 14, 15 reveals the effect of awareness in preventing tuberculosis infection amongst the group of susceptibles with high awareness level as we vary the infection modification parameter σ, from 0 to 1. Of course, a powerful awareness campaign with high awareness rate and with σ = 0, brings about a drop in the proportion of infected individuals especially with an impressive awareness level (e.g., ψ = 30) and less loss of awareness (θ_1_ = θ_2_ = 1).

Clearly, focusing on awareness programmes (for both susceptible and latently infected individuals) is very beneficial for TB control. This should be taken in collaboration with other interventions, like the use of chronic cough as a marker for identifying potential TB cases, improved active case-finding strategy and reduced cost of disease management as well as prolonged awareness programmes to prevent loss of awareness over time.

It is important to state here that the parameter values used in the simulations can be sensitive to the model (3) and there could be uncertainties in their values. However, the use of these parameter values is to demonstrate their effect on the system and gain some insight into the quantitative (especially asymptotic) behaviors of the model.

**Remark**: Note the difference in the time window for the different figures, some having 10, 20, or 100 years of simulation. The graphs having a 10 or 20 year time window was used to observe the pattern of the dynamics of the diseases for the first few years of simulations or disease outbreak. We observe that the results could easily be drowned out if the graphs should have a longer time window. However, the figures with 100 year time window was used to investigate the existence of equilibria based on the parameter values used. It is important to state that tuberculosis dynamics are slow, and consequently, TB epidemics unfold over several decades (Aparicio and Castillo-Chavez, 2009). Hence determining asymptotic behaviors of the disease model could require running simulations spanning a long period of time.

## 4. Discussions and conclusions

A modified and realistic deterministic mathematical model for the transmission dynamics of tuberculosis in a population has been designed and mathematically analyzed. The model (3) extends that formulated and studied in Okuonghae and Omosigho (2011) by classifying both susceptible and latently infected individuals by their level of awareness of tuberculosis (what the disease is, signs and symptoms of tuberculosis and government policy on testing and medical treatment) and included an active case-finding parameter in addition to the use of chronic cough as a marker for identifying potential TB cases, for the control of tuberculosis in a population.

The model (3) has a locally asymptotically stable DFE whenever the associated effective reproduction number is less than one. However, for the treatment free model with insignificant disease-induced death rate, it was shown that the system will undergo the phenomenon of backward bifurcation where the stable disease-free equilibrium will coexist with a stable endemic equilibrium when the effective reproduction number is less than one. For this special case, this phenomenon was caused by the exogenous re-infection of latently infected individuals. We then showed that the effect of exogenous re-infection on the backward bifurcation phenomenon depended strongly on the level of awareness of the latently infected individuals; backward bifurcation due to exogenous re-infection was largely sustained by the latently infected individuals with a low awareness level.

The study further showed that concentrating on TB treatment alone may not significantly reduce the value of the reproduction number if attention is not paid to other key control parameters such as awareness levels and active case-finding rate; in fact, it is possible for the value of the reproduction number to still be greater than one when we concentrate mainly on treatment rate. However, if we take two or more key parameters at the same time and glean out effective control strategies from their combination, then it is possible that, in the long run, the disease burden in the community can be reduced.

Qualitative study of the effective reproduction number showed how different control scenarios involving awareness levels, loss of awareness over time, active case-finding strategy, chronic cough identification and minimal (or no) cost incurred for TB management could lead to a reduction in the value of the effective reproduction number R_*T*_. The analyses suggested that concentrating on increasing tuberculosis awareness campaign for **both** susceptible and latently infected individuals and also increasing chronic cough identification and active case-finding rates will result in reducing the incidence of TB in the population in the presence of an effective cost factor whereby the cost of conducting TB tests and commencing treatment is very small, if not free.

Numerical simulations suggested practical preventive measures, represented by changing the value of some parameters, notably, ν, α_1_, α_2_, θ_1_, θ_2_, ψ, and π. These simulations showed that improving on the cost factor, increased awareness programmes (especially as it affects susceptible and latently infected individuals), cough identification rate as well as minimizing new TB infections caused by fairly isolated TB infectious individuals that are undergoing treatment, will help in reducing the prevalence of TB in the community, together with minimal loss of awareness from both susceptible and latently infected individuals.

In summary, this work have shown, that the prospect of effectively controlling the spread of tuberculosis in a population is very bright. Preventive measures through the use of control strategies should concentrate on awareness programmes. The enlightenment programmes should include helping the general public make use of simple signs in quickly identifying a likely TB case such as chronic cough lasting at least 2 weeks. This will not only quicken the treatment of the infectious individuals, it can also reduce the likelihood of disease transmission. Also awareness programmes should be sustained over a long period of time (especially in places with high endemic levels of tuberculosis) as this work has demonstrated the effect of loss of awareness on the dynamics of tuberculosis in the population.

## Author contributions

Both authors were involved in the discussions and formulation of the mathematical model. BI was involved in the derivation of the effective reproduction number and the backward bifurcation analysis, for the special case considered. DO carried out the analysis of the effective reproduction number, bifurcation analysis for the complete model, as well as the numerical simulations in the manuscript. Both authors were involved in the write-up as well as proof reading of the final draft of the manuscript.

### Conflict of interest statement

The authors declare that the research was conducted in the absence of any commercial or financial relationships that could be construed as a potential conflict of interest.

## Appendix A

### Proofs of theorems 2.1 and 2.2

**Proof of Theorem 2.1**:

*Proof.* Let *t*_1_ = *sup*{*t* > 0 : *S*_1_(*t*) > 0, *S*_2_(*t*) > 0, *E*_1_(*t*) > 0, *E*_2_(*t*) > 0, *I*(*t*) > 0, *J*(*t*) > 0, *T*(*t*) > 0} > 0. It follows from (3a) that

$$\frac{dS_{1}}{dt}=\Lambda -\alpha_{1}S_{1}-\lambda S_{1}-\mu S_{1}+\theta_{1}S_{2}\geq \Lambda -\alpha_{1}S_{1}-\lambda S_{1}-\mu S_{1},$$

which can be written as

$$\frac{d}{dt}\{S_{1}(t)exp[(\alpha_{1}+\mu )t+\int_{0}^{t}\lambda (\tau )d\tau ]\;\}\geq \Lambda \{exp[(\alpha_{1}+\mu )t+\int_{0}^{t}\lambda (\tau )d\tau ]\;\}.$$

Thus,

$$S_{1}(t_{1})exp[(\alpha_{1}+\mu )t_{1}+\int_{0}^{t_{1}}\lambda (\tau )d\tau ]-S_{1}(0)\geq \int_{0}^{t_{1}}\Lambda \{exp[(\alpha_{1}+\mu )y+\int_{0}^{y}\lambda (\tau )d\tau ]\;\}dy.$$

so that,

$$\begin{array}{l} S_{1}(t_{1})\geq S_{1}(0)exp\left[-(\alpha_{1}+\mu )t_{1}-\int_{0}^{t_{1}}\lambda (\tau )d\tau \right]+\\ \;-\left\{exp\left[-(\alpha_{1}+\mu )t_{1}-\int_{0}^{t_{1}}\lambda (\tau )d\tau \right]\;\right\}\\ \;\int_{0}^{t_{1}}\Lambda \left\{exp\left[(\alpha_{1}+\mu )y+\int_{0}^{y}\lambda (\tau )d\tau \right]\;\right\}dy>0. \end{array}$$

Similarly, it can be shown that *S*_2_(*t*) > 0, *E*_1_(*t*) > 0, *E*_2_(*t*) > 0, *I*(*t*) > 0, *J*(*t*) > 0 and *T*(*t*) > 0 for all time *t* > 0. Hence all solutions of the model (3) remain positive for all non-negative initial conditions, as required.        □

**Proof of Theorem** **2.2**:

*Proof.* Adding the equations of the model (3) gives

(A1) $$\frac{dN}{dt}=\Lambda -\mu N-dI.$$

Since the right hand side of the above equality is bounded by Λ − μ*N*, a standard comparison theorem (Lakshmikantham et al., 1989) can be used to show that

(A2) $$N(t)\leq N(0)e^{-\mu t}+\frac{\Lambda }{\mu }(1-e^{-\mu t}).$$

In particular, if $N\left(0\right)\leq \frac{\Lambda }{\mu }$, then $N\left(t\right)\leq \frac{\Lambda }{\mu }$. Thus, D is a positively invariant set. Furthermore, if $N\left(0\right)\geq \frac{\Lambda }{\mu }$, then either the solution enters D in finite time or *N*(*t*) approaches to $\frac{\Lambda }{\mu }$ as *t* → ∞. Hence no solution path leave through any boundary of D and the region D attracts all solutions in ${\mathbb{R}}_{+}^{7}$.           □

## Appendix B

### Partial derivatives of R_*T*_

(A3) $$\frac{\partial R_{T}}{\partial r}=-\frac{\beta \eta (\pi +\nu \alpha_{2})P_{1}}{\left({\left(\mu +r\right)}^{2}(d+\pi +\mu +\nu \alpha_{2})(\mu +\alpha_{1}+\theta_{1})((\mu +k_{1})(\mu +k_{2}+\theta_{2})+(\mu +k_{2})\psi )\right)},$$

(A4) $$\frac{\partial R_{T}}{\partial \alpha_{1}}=\frac{\beta (\pi \eta +\mu +r+\eta \nu \alpha_{2})(\mu +\theta_{1})P_{2}}{\left((\mu +r)(d+\pi +\mu +\nu \alpha_{2}){\left(\mu +\alpha_{1}+\theta_{1}\right)}^{2}\;((\mu +k_{1})(\mu +k_{2}+\theta_{2})+(\mu +k_{2})\psi )\right)},$$

(A5) $$\frac{\partial R_{T}}{\partial \theta_{1}}=-\frac{\beta \alpha_{1}(\pi \eta +\mu +r+\eta \nu \alpha_{2})P_{2}}{\left((\mu +r)(d+\pi +\mu +\nu \alpha_{2}){\left(\mu +\alpha_{1}+\theta_{1}\right)}^{2}\;((\mu +k_{1})(\mu +k_{2}+\theta_{2})+(\mu +k_{2})\psi )\right)},$$

(A6) $$\frac{\partial R_{T}}{\partial \theta_{2}}=\frac{\beta \mu (\pi \eta +\mu +r+\eta \nu \alpha_{2})(k_{1}-k_{2})[(1-p_{1})(\mu +\theta_{1})\psi +\sigma (1-p_{2})\alpha_{1}(\mu +k_{1}+\psi )]}{\left((\mu +r)(d+\pi +\mu +\nu \alpha_{2})(\mu +\alpha_{1}+\theta_{1}){\left((\mu +k_{1})(\mu +k_{2}+\theta_{2})+(\mu +k_{2})\psi \right)}^{2}\right)},$$

(A7) $$\frac{\partial R_{T}}{\partial \psi }=\frac{\beta \mu (\pi \eta +\mu +r+\eta \nu \alpha_{2})(k_{1}-k_{2})[(1-p_{1})(\mu +\theta_{1})(\mu +k_{2}+\theta_{2})+\sigma (1-p_{2})\alpha_{1}\theta_{1}]}{\left((\mu +r)(d+\pi +\mu +\nu \alpha_{2})(\mu +\alpha_{1}+\theta_{1}){\left((\mu +k_{1})(\mu +k_{2}+\theta_{2})+(\mu +k_{2})\psi \right)}^{2}\right)},$$

(A8) $$\frac{\partial R_{T}}{\partial \pi }=\frac{\beta (\eta d-(1-\eta )\mu -r)P_{1}}{\left((\mu +r){\left(d+\pi +\mu +\nu \alpha_{2}\right)}^{2}(\mu +\alpha_{1}+\theta_{1})((\mu +k_{1})(\mu +k_{2}+\theta_{2})+(\mu +k_{2})\psi )\right)},$$

(A9) $$\frac{\partial R_{T}}{\partial \alpha_{2}}=\frac{\beta \nu (\eta d-(1-\eta )\mu -r)P_{1}}{\left((\mu +r){\left(d+\pi +\mu +\nu \alpha_{2}\right)}^{2}(\mu +\alpha_{1}+\theta_{1})((\mu +k_{1})(\mu +k_{2}+\theta_{2})+(\mu +k_{2})\psi )\right)},$$

(A10) $$\frac{\partial R_{T}}{\partial \nu }=\frac{\beta \alpha_{2}(\eta d-(1-\eta )\mu -r)P_{1}}{\left((\mu +r){\left(d+\pi +\mu +\nu \alpha_{2}\right)}^{2}(\mu +\alpha_{1}+\theta_{1})((\mu +k_{1})(\mu +k_{2}+\theta_{2})+(\mu +k_{2})\psi )\right)},$$

where

*P*_1_ = *k*_1_(μ(μ + σα_1_*p*_2_ + θ_1_ + (μ + σα_1_ + θ_1_)(*k*_2_ + θ_2_)) + σα_1_*k*_2_(μ + ψ) + *k*_2_(μ + θ_1_)(μ*p*_1_ + ψ) + μ(σα_1_*p*_2_ + *p*_1_(μ + θ_1_))(μ + θ_2_ + ψ)),

and

*P*_2_ = −[(1 − σ)(*k*_1_*k*_2_ + *k*_1_θ_2_ + *k*_2_ψ) + *k*_1_μ(1 − σ*p*_2_)] − (*k*_2_μ(*p*_1_ − σ) + μ(*p*_1_ − σ*p*_2_)(μ + θ_2_ + ψ).

## Appendix C

### Existence of EEP for special case

The coefficients of the polynomial (25) are given as

$$\begin{array}{l} A_{1}=z^{2}\vartheta \sigma \Lambda (\mu +\pi +\nu \alpha_{2}),\\ A_{2}=\Lambda z(\mu +\pi +\nu \alpha_{2})[\mu (\sigma +\vartheta (z+\sigma +z\sigma ))+\vartheta \sigma k_{1}+\sigma k_{2}+z\vartheta \sigma \alpha_{1}\\ \;+z\vartheta \theta_{1}+\sigma (\theta_{2}+\vartheta \psi )]-[\beta (\mu +\eta (\pi +\nu \alpha_{2}))z^{2}\vartheta \Lambda \sigma ]\\ A_{3}=\Lambda (\mu +\pi +\nu \alpha_{2})[k_{1}(\mu (\sigma +z\vartheta (1+\sigma ))+\sigma k_{2}+z\vartheta (\sigma \alpha_{1}+\theta_{1})+\sigma \theta_{2})\\ \;+z\theta_{1}((1+\vartheta +z\vartheta )\mu +\theta_{2}+\vartheta \psi )+k_{2}(\mu (z+\sigma +z\sigma )+z\sigma \alpha_{1}+z\theta_{1}+\sigma \psi )\\ \;+z\alpha_{1}(\mu (\sigma +\vartheta (z+\sigma ))+\sigma \theta_{2}+\vartheta \sigma \psi )+\mu [\mu (\sigma +z(1+\sigma +\vartheta (1+z+\sigma )))\\ \;+(z+\sigma +z\sigma )\theta_{2}(\sigma +z\vartheta (1+\sigma ))\psi ]]-[\beta (\mu +\eta (\pi +\nu \alpha_{2}))\\ \;z\Lambda (z\vartheta \mu +\mu \sigma +\vartheta \sigma k_{1}+\sigma k_{2}+\vartheta \mu \sigma p_{1}+z\vartheta \sigma \alpha_{1}+z\vartheta \theta_{1}+\sigma \theta_{2}+\vartheta \sigma \psi )]\\ A_{4}=\Lambda (\mu +\pi +\nu \alpha_{2})[k_{1}[k_{2}(\mu +\mu \sigma +\sigma \alpha_{1}+\theta_{1})+\theta_{1}(\mu +z\vartheta \mu +\theta_{2})+\alpha_{1}(\mu (z\vartheta +\sigma )+\sigma \theta_{2})\\ \;+\mu (\mu (1+z\vartheta +\sigma )+(1+\sigma )\theta_{2})]+k_{2}[\theta_{1}(\mu +z\mu +\psi_{1})+\alpha_{1}(\mu (z+\sigma )+\sigma \psi )\\ \;+\mu (\mu (1+z+\sigma )+(1+\sigma )\psi )]+\mu [\theta_{1}((1+z+z\vartheta )\mu +(1+z)\theta_{2}+(1+z\vartheta )\psi )\\ \;+\alpha_{1}(\mu (z+z\vartheta +\sigma )+(z+\sigma )\theta_{2}+(z\vartheta +\sigma )\psi )+\mu (\mu (1+z+z\vartheta +\sigma )\\ \;+(1+z+\sigma )\theta_{2}+(1+z\vartheta +\sigma )\psi )]\\ \;-\beta (\mu +\eta (\pi +\nu \alpha_{2}))\Lambda [k_{1}(\mu (z\vartheta +\sigma )+\sigma k_{2}+z\vartheta (\sigma \alpha_{1}+\theta_{1})+\sigma \theta_{2})\\ \;+k_{2}(\mu \sigma p_{1}+z(\mu +\sigma \alpha_{1}+\theta_{1})+\sigma \psi )+\mu p_{1}(\mu (z\vartheta +\sigma )+z\vartheta \theta_{1}+\sigma (\theta_{2}+\psi ))\\ \;z((\mu +\theta_{1})(\mu +\theta_{2}+\vartheta \psi )+\sigma \alpha_{1}(\mu np_{2}+\theta_{2}+\vartheta (\mu +\psi )))]\\ A_{5}=\Lambda \mu (\mu +\alpha_{1}+\theta_{1})(\mu +\pi +\nu \alpha_{2})((\mu +k_{1})(\mu +k_{2}+\theta_{2})+(\mu +k_{2})\psi )[1-{\hat{R}}_{T}] \end{array}$$

where *z* = *b*_1_ and ϑ = *b*_1_*b*_2_ with ${\hat{R}}_{T}=R_{T}{|}_{r=d=0}$ being the reproduction number of the model (3) where *r* = *d* = 0. The endemic steady state solutions are

(A11) $$\begin{array}{l} S_{1}^{**}=\frac{\Lambda (\mu +\lambda^{e}\sigma +\theta_{1})}{\alpha_{1}(\mu +\lambda^{e}\sigma )+(\lambda^{e}+\mu )(\mu +\lambda^{e}\sigma +\theta_{1})},\\ S_{2}^{**}=\frac{\Lambda \alpha_{1}}{\alpha_{1}(\mu +\lambda^{e}\sigma )+(\lambda^{e}+\mu )(\mu +\lambda^{e}\sigma +\theta_{1})},\\ E_{1}^{**}=\frac{1}{b_{1}\lambda^{e}+(k_{1}+\mu +\psi )}[(1-p_{1})S_{1}^{**}\lambda^{e}+\theta_{2}E_{2}^{**}],\\ E_{2}**=\frac{1}{b_{2}\lambda^{e}+(k_{2}+\mu +\theta_{2})}[(1-p_{2})\sigma S_{2}^{**}\lambda^{e}+\psi E_{1}^{**}],\\ I^{**}=\frac{1}{\nu \alpha_{2}+\mu +\pi }[p_{1}S_{1}^{**}\lambda^{e}+p_{2}\sigma S_{2}^{**}\lambda^{e}+(b_{1}E_{1}^{**}+b_{2}E_{2}^{**})+k_{1}E_{1}^{**}+k_{2}E_{2}^{**}],\\ J^{**}=\frac{\pi +\nu \alpha_{2}}{\mu }I^{**} \end{array}$$

## Appendix D

### Proof of theorem 2.4

Suppose

$$\xi_{3}=(S_{1}^{**},S_{2}^{**},E_{1}^{**},E_{2}^{**},I^{**},J^{**},T^{**})$$

represents any arbitrary endemic equilibrium of model (3) (i.e., an equilibrium in which at least one of the infected components is non-zero). We will explore the existence of backward bifurcation using the center manifold theory (Carr, 1981; Castillo-Chavez and Song, 2004). To apply this theory, it is convenient to perform the following change of variables. Let *S*_1_ = *x*_1_, *S*_2_ = *x*_2_, *E*_1_ = *x*_3_, *E*_2_ = *x*_4_, *I* = *x*_5_, *J* = *x*_6_, and *T* = *x*_7_. Further, by using the vector notation $X={\left(x_{1},x_{2},\ldots ,x_{7}\right)}^{T}$, and noting that the force of infection is given by

$$\lambda =\beta \frac{x_{5}+\eta x_{6}}{x_{1}+x_{2}+x_{3}+x_{4}+x_{5}+x_{6}+x_{7}},$$

the model (3) can be written in the form $\frac{dX}{dt}=F\left(X\right)$, with $F={\left(f_{1},f_{2},\ldots ,f_{7}\right)}^{T}$, as follows

(A12) $$\begin{array}{l} \dot{x_{1}}\equiv f_{1}=\Lambda -\alpha_{1}x_{1}-\lambda x_{1}+\theta_{1}x_{2}-\mu x_{1},\\ \dot{x_{2}}\equiv f_{2}=\alpha_{1}x_{1}-\sigma \lambda x_{2}-\theta_{1}x_{2}-\mu x_{1},\\ \dot{x_{3}}\equiv f_{3}=(1-p_{1})\lambda (x_{1}+\omega \epsilon x_{7})-b_{1}\lambda x_{3}-(k_{1}+\psi_{1}+\mu )x_{3}+\theta_{2}x_{4},\\ \dot{x_{4}}\equiv f_{4}=(1-p_{2})\lambda (\sigma x_{2}+(1-\omega )\epsilon x_{7})-b_{2}\lambda x_{4}-(k_{2}+\theta_{2}+\mu )x_{4}+\psi_{1}x_{3},\\ \dot{x_{5}}\equiv f_{5}=p_{1}\lambda (x_{1}+\omega \epsilon x_{7})+p_{2}\lambda (\sigma x_{2}+(1-\omega )\epsilon x_{7})+b_{1}\lambda x_{3}+b_{2}\lambda x_{4}\\ \;+k_{1}x_{3}+k_{2}x_{4}-(\nu \alpha +\pi +d+\mu )x_{5},\\ \dot{x_{6}}\equiv f_{6}=\pi x_{5}+\nu \alpha_{2}x_{5}-(r_{2}+\mu )x_{6},\\ \dot{x_{7}}\equiv f_{7}=r_{2}x_{6}-\epsilon \lambda x_{7}-\mu x_{7}. \end{array}$$

Consider the case when β = β^*^ is chosen as a bifurcation parameter. Solving for β = β^*^ from R_*T*_ = 1 gives

$$\beta =\beta^{*}=\frac{G_{4}}{\left(\mu +\eta \pi +r_{2}+\eta \nu \alpha_{2})(G_{1}+G_{2}+G_{3}\right)}.$$

The Jacobian of the transformed system (3), evaluated at the disease-free equilibrium (ξ_1_) with β = β^*^, is given by

$$J^{*}=J(\xi_{1}){|}_{\beta =\beta^{*}}=\left(\begin{array}{ccccccc} -K_{1} & \theta_{1} & 0 & 0 & -\frac{\beta^{*}x_{1}^{*}}{x_{1}^{*}+x_{2}^{*}} & -\frac{\eta \beta^{*}x_{1}^{*}}{x_{1}^{*}+x_{2}^{*}} & 0\\ \alpha_{1} & -K_{2} & 0 & 0 & -\frac{\sigma \beta^{*}x_{2}^{*}}{x_{1}^{*}+x_{2}^{*}} & -\frac{\sigma \eta \beta^{*}x_{2}^{*}}{x_{1}^{*}+x_{2}^{*}} & 0,\\ 0 & 0 & -K_{3} & \theta_{2} & \frac{(1-p_{1})\beta^{*}x_{1}^{*}}{x_{1}^{*}+x_{2}^{*}} & \frac{(1-p_{1})\eta \beta^{*}x_{1}^{*}}{x_{1}^{*}+x_{2}^{*}} & 0,\\ 0 & 0 & \psi & -K_{4} & \frac{(1-p_{2})\sigma \beta^{*}x_{2}^{*}}{x_{1}^{*}+x_{2}^{*}} & \frac{(1-p_{2})\sigma \eta \beta^{*}x_{2}^{*}}{x_{1}^{*}+x_{2}^{*}} & 0,\\ 0 & 0 & k_{1} & k_{2} & \beta \frac{p_{1}x_{1}^{*}+p_{2}\sigma x_{2}^{*}}{x_{1}^{*}+x_{2}^{*}}-K_{5} & \beta \eta \frac{p_{1}x_{1}^{*}+p_{2}\sigma x_{2}^{*}}{x_{1}^{*}+x_{2}^{*}} & 0,\\ 0 & 0 & 0 & 0 & \pi +\nu \alpha_{2} & -K_{6} & 0,\\ 0 & 0 & 0 & 0 & 0 & r_{2} & -\mu , \end{array}\right)$$

where *K*_1_ = μ + α_1_, *K*_2_ = θ_1_ + μ, *K*_3_ = *k*_1_ + ψ + μ, *K*_4_ = *k*_2_ + θ_2_ + μ, *K*_5_ = να_2_ + μ + *d* + π, *K*_6_ = *r*_2_ + μ, $x_{1}^{*}=\frac{\Lambda }{\mu }\frac{\mu +\theta_{1}}{\mu +\alpha_{1}+\theta_{1}}$ and $x_{2}^{*}=\frac{\Lambda }{\mu }\frac{\alpha_{1}}{\mu +\alpha_{1}+\theta_{1}}$.

Matrix *J*^*^ has a right eigenvector given by $w={\left(\omega_{1},\omega_{2},\ldots ,\omega_{11}\right)}^{T}$, where

(A13) $$\begin{array}{l} \omega_{1}=-\frac{G_{4}(\mu^{2}+\theta_{1}(2\mu +\sigma \alpha_{1}+\theta_{1}))\omega_{5}}{\mu (G_{1}+G_{2}+G_{3})(\mu +r){\left(\mu +\alpha_{1}+\theta_{1}\right)}^{2}}\equiv -H_{1}w_{5}<0\\ \omega_{2}=-\frac{G_{4}\alpha_{1}(\mu +\sigma \mu +\sigma \alpha_{1}+\theta_{1})\omega_{5}}{\mu (G_{1}+G_{2}+G_{3})(\mu +r){\left(\mu +\alpha_{1}+\theta_{1}\right)}^{2}}\equiv -H_{2}w_{5}<0\\ \omega_{3}=\frac{G_{4}[(1-p_{1})(\mu +\theta_{1})(\mu +k_{2}+\theta_{2})+\sigma \alpha_{1}\theta_{2}(1-p_{2})]\omega_{5}}{\left(G_{1}+G_{2}+G_{3})(\mu +r)(\mu +\alpha_{1}+\theta_{1})[(\mu +k_{1})(\mu +k_{2}+\theta_{2})+(\mu +k_{2})\psi \right]}\equiv H_{3}\omega >0,\\ \omega_{4}=\frac{G_{4}[(1-p_{1})(\mu +\theta_{1})\psi +\sigma (1-p_{2})\alpha_{1}(\mu +k_{1}+\psi )]\omega_{5}}{\left(G_{1}+G_{2}+G_{3})(\mu +r)(\mu +\alpha_{1}+\theta_{1})[(\mu +k_{1})(\mu +k_{2}+\theta_{2})+(\mu +k_{2})\psi \right]}\equiv H_{4}\omega >0,\\ \omega_{5}=\omega_{5}>0,\\ \omega_{6}=\frac{\pi +\nu \alpha_{2}}{r+\nu }\omega_{5}\equiv H_{5}\omega_{5}>0,\\ \omega_{7}=\frac{r(\pi +\nu \alpha_{2})}{r+\nu }\omega_{5}\equiv H_{6}\omega_{5}>0. \end{array}$$

Furthermore, *J*^*^ has a left eigenvector *v* = (ν_1_, ν_2_, …., ν_11_) satisfying **v.w** = **1**, with

(A14) $$\begin{array}{l} \nu_{1}=\frac{\alpha_{1}}{\left(\alpha_{1}+\mu )(\theta_{1}+\mu \right)}\equiv Z_{1}>0,\\ \nu_{2}=\frac{\alpha_{1}+\mu }{\theta_{1}\alpha_{1}}\equiv Z_{2}>0,\\ \nu_{3}=\frac{k_{1}(\mu +k_{2}+\theta_{2})+k_{2}\psi }{(\mu +k_{1})(\mu +k_{2}+\theta_{2})+(\mu +k_{2})\psi }\nu_{5}\equiv Z_{3}\nu_{5},\\ \nu_{4}=\frac{k_{1}\theta_{2}+k_{2}(\mu +k_{1}+\psi )}{(\mu +k_{1})(\mu +k_{2}+\theta_{2})+(\mu +k_{2})\psi }\nu_{5}\equiv Z_{4}\nu_{5},\\ \nu_{5}=\nu_{5}>0,\\ \nu_{6}=\frac{1}{r+\mu }[-\frac{\eta \beta^{*}x_{1}^{*}}{x_{1}^{*}+x_{2}^{*}}\nu_{1}-\frac{\sigma \eta \beta^{*}x_{2}^{*}}{x_{1}^{*}+x_{2}^{*}}\nu_{2}+\frac{(1-p_{1})\eta \beta^{*}x_{1}^{*}}{x_{1}^{*}+x_{2}^{*}}\nu_{3}\\ \;+\frac{(1-p_{2})\sigma \eta \beta^{*}x_{2}^{*}}{x_{1}^{*}+x_{2}^{*}}\nu_{4}+\beta \eta \frac{p_{1}x_{1}^{*}+p_{2}\sigma x_{2}^{*}}{x_{1}^{*}+x_{2}^{*}}\nu_{5}],\\ \nu_{7}=0. \end{array}$$

It follows from Theorem 4.1 in Castillo-Chavez and Song (2004), if we compute the associated non-zero partial derivatives of F(x) (evaluated at the DFE, ξ_1_), that the associated bifurcation coefficients, *a* and *b*, defined by

$$a=\sum_{k,i,j=1}^{n}v_{k}w_{i}w_{j}\frac{\partial^{2}f_{k}}{\partial x_{i}\partial x_{j}}(0,0),$$

and

$$b=\sum_{k,i=1}^{n}v_{k}w_{i}\frac{\partial^{2}f_{k}}{\partial x_{i}\partial \beta^{*}}(0,0),$$

are computed to be

(A15) $$\begin{array}{l} a=-\frac{2\beta^{*}\omega_{5}^{2}(1+\eta H_{5})}{x_{1}^{*}+x_{2}^{*}}\{(H_{1}+H_{2})\left[\frac{x_{1}^{*}}{x_{1}^{*}+x_{2}^{*}}Z_{1}+\frac{\sigma x_{2}^{*}}{x_{1}^{*}+x_{2}^{*}}Z_{2}\right]\\ \;+(H_{3}+H_{4}+H_{5}+H_{6}+1)\left[\frac{(1-p_{1})x_{1}^{*}}{x_{1}^{*}+x_{2}^{*}}Z_{3}+\frac{\sigma (1-p_{2})x_{2}^{*}}{x_{1}^{*}+x_{2}^{*}}Z_{4}+\frac{p_{1}x_{1}^{*}}{x_{1}^{*}+x_{2}^{*}}+\frac{\sigma p_{2}x_{2}^{*}}{x_{1}^{*}+x_{2}^{*}}\right]\nu_{5}\\ \;+\left[(1-p_{1})H_{1}Z_{3}+b_{1}H_{3}Z_{3}+\sigma (1-p_{2})H_{2}Z_{4}+b_{2}H_{4}Z_{4}+\frac{p_{1}}{2}H_{1}+\frac{\sigma p_{2}}{2}H_{2}\right]\nu_{5}\\ \;-((H_{1}+H_{2})\left[\frac{(1-p_{1})x_{1}^{*}}{x_{1}^{*}+x_{2}^{*}}Z_{3}+\frac{\sigma (1-p_{2})x_{2}^{*}}{x_{1}^{*}+x_{2}^{*}}Z_{4}+\frac{p_{1}x_{1}^{*}}{x_{1}^{*}+x_{2}^{*}}+\frac{\sigma p_{2}x_{2}^{*}}{x_{1}^{*}+x_{2}^{*}}\right]\nu_{5}\\ \;+(H_{3}+H_{4}+H_{5}+H_{6}+1)\left[\frac{x_{1}^{*}}{x_{1}^{*}+x_{2}^{*}}Z_{1}+\frac{\sigma x_{2}^{*}}{x_{1}^{*}+x_{2}^{*}}Z_{2}\right]\\ \;+H_{1}Z_{1}+\sigma H_{2}Z_{2}+[\epsilon \omega (1-p_{1})H_{6}Z_{3}+\epsilon (1-\omega )(1-p_{2})H_{6}Z_{4}\\ \;+\frac{b_{1}}{2}H_{3}+\frac{b_{2}}{2}H_{4}+\epsilon \omega p_{1}H_{6}+\epsilon (1-\omega )p_{2}H_{6}]\nu_{5})\} \end{array}$$

and

(A16) $$b=\left(Z_{3}\frac{(1-p_{1})x_{1}^{*}}{x_{1}^{*}+x_{2}^{*}}+Z_{4}\frac{(1-p_{2})\sigma x_{2}^{*}}{x_{1}^{*}+x_{2}^{*}}+\frac{p_{1}x_{1}^{*}}{x_{1}^{*}+x_{2}^{*}}+\frac{p_{2}x_{2}^{*}}{x_{1}^{*}+x_{2}^{*}}\right)v_{5}-Z_{1}\frac{x_{1}^{*}}{x_{1}^{*}+x_{2}^{*}}+Z_{2}\frac{\sigma x_{2}^{*}}{x_{1}^{*}+x_{2}^{*}},$$

for *v*_5_ > 0.

It follows from Theorem 4.1 in Castillo-Chavez and Song (2004) that the model (3), or the transformed model (A12), will undergo a backward bifurcation if the backward bifurcation coefficients *a* and *b*, given by (A15) and (A16), are positive. However, if *a* is negative, with *b* still remaining positive, then the system [either (3) or (A12)] will not undergo a backward bifurcation.             □
